# *Aldh1l2* knockout mouse metabolomics links the loss of the mitochondrial folate enzyme to deregulation of a lipid metabolism observed in rare human disorder

**DOI:** 10.1186/s40246-020-00291-3

**Published:** 2020-11-09

**Authors:** Natalia I. Krupenko, Jaspreet Sharma, Peter Pediaditakis, Kristi L. Helke, Madeline S. Hall, Xiuxia Du, Susan Sumner, Sergey A. Krupenko

**Affiliations:** 1 Nutrition Research Institute, University of North Carolina, Chapel Hill, NC USA; 2 Department of Nutrition, University of North Carolina, Chapel Hill, NC USA; 3 Department of Comparative Medicine, Medical University of South Carolina, Charleston, SC USA; 4 Department of Bioinformatics & Genomics, UNC Charlotte, Charlotte, NC USA

**Keywords:** ALDH1L2, Folate metabolism, Knockout mouse model, Metabolomics, NADPH, Oxidative stress, β-oxidation, Coenzyme A

## Abstract

**Background:**

Mitochondrial folate enzyme ALDH1L2 (aldehyde dehydrogenase 1 family member L2) converts 10-formyltetrahydrofolate to tetrahydrofolate and CO_2_ simultaneously producing NADPH. We have recently reported that the lack of the enzyme due to compound heterozygous mutations was associated with neuro-ichthyotic syndrome in a male patient. Here, we address the role of ALDH1L2 in cellular metabolism and highlight the mechanism by which the enzyme regulates lipid oxidation.

**Methods:**

We generated *Aldh1l2* knockout (KO) mouse model, characterized its phenotype, tissue histology, and levels of reduced folate pools and applied untargeted metabolomics to determine metabolic changes in the liver, pancreas, and plasma caused by the enzyme loss. We have also used NanoString Mouse Inflammation V2 Code Set to analyze inflammatory gene expression and evaluate the role of ALDH1L2 in the regulation of inflammatory pathways.

**Results:**

Both male and female *Aldh1l2* KO mice were viable and did not show an apparent phenotype. However, H&E and Oil Red O staining revealed the accumulation of lipid vesicles localized between the central veins and portal triads in the liver of *Aldh1l2*^*-/-*^ male mice indicating abnormal lipid metabolism. The metabolomic analysis showed vastly changed metabotypes in the liver and plasma in these mice suggesting channeling of fatty acids away from β-oxidation. Specifically, drastically increased plasma acylcarnitine and acylglycine conjugates were indicative of impaired β-oxidation in the liver. Our metabolomics data further showed that mechanistically, the regulation of lipid metabolism by ALDH1L2 is linked to coenzyme A biosynthesis through the following steps. ALDH1L2 enables sufficient NADPH production in mitochondria to maintain high levels of glutathione, which in turn is required to support high levels of cysteine, the coenzyme A precursor. As the final outcome, the deregulation of lipid metabolism due to ALDH1L2 loss led to decreased ATP levels in mitochondria.

**Conclusions:**

The ALDH1L2 function is important for CoA-dependent pathways including β-oxidation, TCA cycle, and bile acid biosynthesis. The role of ALDH1L2 in the lipid metabolism explains why the loss of this enzyme is associated with neuro-cutaneous diseases. On a broader scale, our study links folate metabolism to the regulation of lipid homeostasis and the energy balance in the cell.

**Supplementary Information:**

The online version contains supplementary material available at 10.1186/s40246-020-00291-3.

## Background

Folate metabolism, linked to the one-carbon transfer, supports fundamental cellular processes such as amino acid biogenesis, nucleotide biosynthesis, methylation, and NADPH production [1]. Biochemical reactions involving folate coenzymes are executed by more than 20 enzymes, which typically reside in cytoplasm or mitochondria [1]. ALDH1L2, a mitochondrial enzyme in folate metabolism, converts 10-formyl-THF (tetrahydrofolate) to THF and CO_2_ in a NADP^+^-dependent reaction [2]. While the biological significance of this enzyme is not fully understood, it was reported that the ALDH1L2-catalyzed reaction is an important source of NADPH in mitochondria [3], and this function links the enzyme to melanoma metastasis [4]. We have recently reported that the loss of the ALDH1L2 activity through deleterious mutations in the *ALDH1L2* gene is linked to neuro-ichthyotic syndrome [5]. Such syndromes represent a group of rare genetic diseases commonly associated with impaired lipid metabolism [6]. One of the most recognized of these conditions, Sjögren–Larsson syndrome (SLS: MIM#270200), is typically caused by mutations in the *ALDH3A2* gene encoding for fatty acid aldehyde dehydrogenase and characterized by congenital ichthyosis, leukoencephalopathy, intellectual disability, and spastic di- or tetraplegia [7]. ALDH1L2-deficient patient displayed atypical phenotype with fibroblasts derived from his skin showing strong changes in acylcarnitines and intermediates of the Krebs cycle compared to the cells from healthy individuals [5]. Overall, this study demonstrated that ALDH1L2 is important for the mitochondrial integrity and for maintenance of the energy balance in the cell.

A homologous enzyme with the same activity, ALDH1L1, resides in cytoplasm [8]. The two enzymes are products of different genes with mitochondrial ALDH1L2 appearing as the result of gene duplication during vertebrate evolution; the event can be traced to bony fish [9]. The primordial *Aldh1l1* gene was the product of natural fusion of three unrelated genes, (i) a folate-related formyltransferase, (ii) an acyl carrier protein, and (iii) an aldehyde dehydrogenase [8]. Merging a folate-binding domain with the aldehyde dehydrogenase catalytic machinery enabled a novel enzymatic reaction, the oxidation of folate-bound formyl group to CO_2_. Experimental data suggest that this reaction is important for the regulation of the overall cellular folate pool as well as the proliferative capacity of the cell [10, 11]. We have recently generated mice with targeted *Aldh1l1* knockout and demonstrated that the animals lacking the enzyme display metabolic signs of folate deficiency and have impaired glycine metabolism in the liver [12]. The cause of these metabolic perturbations was the inability, in the absence of ALDH1L1, to convert 10-formyl-THF to THF resulting in the shortage of the latter metabolite required for the generation of glycine from serine [12]. These findings support the hypothesis that in non-proliferating cells the ALDH1L1-catalyzed reaction is important to restore the THF pool [13]. THF is the acceptor of one-carbon groups in the folate metabolism and is required for the serine to glycine conversion, histidine degradation, and formate oxidation, the reactions taking place in cytoplasm [1]. In mitochondria, THF is involved in additional reactions, the glycine degradation and the utilization of the intermediates of choline/betaine metabolism, dimethylglycine, and sarcosine [1].

Mitochondria and cytoplasm cannot freely exchange folate coenzymes or metabolites of folate pathways, the phenomenon causing strict compartmentalization of folate metabolism [1]. Accordingly, two sets of folate enzymes, cytoplasmic and mitochondrial, are present in eukaryotic cells. These enzymes play specialized roles in cellular metabolism but may have somewhat overlapping functions as demonstrated for serine hydroxymethyltransferases [14]. The question of whether ALDH1L1 and ALDH1L2 functions are redundant or whether they have independent significance for the cell remained unanswered. The overlapping function of the two enzymes is perhaps suggested by the fact that birds and reptiles have lost the gene for the cytosolic enzyme [9]. In our previous study, *Aldh1l1* knockout mice were viable and did not have apparent phenotype [12] raising the question of whether the mitochondrial enzyme can compensate for the loss of the cytosolic counterpart. Here, we have generated and characterized *Aldh1l2* knockout mice and demonstrate that in contrast to ALDH1L1, the mitochondrial enzyme is important for the regulation of lipid metabolism.

## Materials and methods

### Generation of *Aldh1l2* knockout mice

All animal experiments were conducted in strict accordance with the National Institutes of Health’s “Guide for Care and Use of Laboratory Animals” and were approved by the Institutional Animal Care and Use Committee at the Medical University of South Carolina (MUSC), Charleston, South Carolina, and by the Institutional Animal Care and Use Committee at the David H. Murdock Research Institute (DHMRI), Kannapolis, North Carolina. Mice were housed in microisolator cages on a 12-h light/dark cycle and allowed access to water and chow ad libitum. ES cells (clone EPD0514_5_F12 with the targeted non-conditional allele for Aldh1l2) were obtained from the NCRR-NIH-supported KOMP Repository (www.komp.org). This clone was from the C57BL/6N background with the agouti gene engineered into the agouti locus. Mice were generated at the MUSC Transgenic and Genome Editing Core Facility by a standard method [15]. ES cells (passage 4) have been expanded and injected into freshly isolated C57BL/6-Tyr^C^ blastocysts (10 ES cells per blastocyte), which were further injected into CD-1 pseudo-pregnant mice (12 blastocysts per mouse). Six chimeric pups were born from three injected females and at weaning, we obtained four male chimeric mice, three having 70–98% and one having 40% of the agouti coat color. All male chimeras were bred to C57BL/6-Tyr^C^ females, and both black and agouti pups (male and female) were tested for germline transmission of the targeted gene. Two of the chimeras (40% and 98%) showed germline transmission. Mice, heterozygous for the targeted allele, were bred back to the C57BL/6N mice. Heterozygous (*Aldh1l2*^+/−^) breeding pairs were used to generate knockout and wild type littermates for the studies.

### Genotyping

Genotyping was carried out by polymerase chain reaction (PCR) of tail lysates obtained using direct PCR (Tail) lysis reagent (cat #101-T) and Proteinase K (specific activity > 600 U/ml, Thermo Scientific, cat #EO0491). Primers for genotyping are shown in Supplementary Table S1.

### Tissue histology

Three-month-old mice were euthanized using CO_2_, organs were harvested, fixed using 4% paraformaldehyde in sodium phosphate buffer pH 7.4, and embedded in paraffin. Five-micrometer sections were deparaffinized, rehydrated, and stained with H&E. For Oil Red O staining, the liver tissues (frozen OCT blocks) were cut into 5 μm sections, mounted on slides, and allowed to dry. The sections were fixed in 10% formalin for 10 min, and then, the slides were rinsed with PBS (PH 7.4). After air dry, the slides were placed in 100% propylene glycol for 2 min and stained in 0.5% Oil Red O solution in propylene glycol for 30 min. The slides were then transferred to an 85% propylene glycol solution for 1 min, rinsed in distilled water twice, and processed for hematoxylin counterstaining.

### Western blot assays

Total protein was prepared from flash-frozen tissues. Approximately 300 mg of the tissue was minced and homogenized in 1 ml of RIPA or IP-lysis buffer (Thermo Scientific, Pittsburg, PA) with protease and phosphatase inhibitors (1:100, Sigma-Aldrich, St. Louis, MO). Proteins were resolved by SDS-PAGE in 4–15% gradient gels (Novex, Invitrogen, Carlsbad, CA) and transferred to PVDF membranes (Millipore, Bedford, MA) in a transfer buffer containing 10% methanol. The membranes were probed with primary antibodies in Tris-buffered saline with Tween-20 and 5% nonfat milk or BSA. A detailed description of the primary antibodies is provided in Supplementary Table S2. Horseradish peroxidase-conjugated secondary antibodies (GE Healthcare) were used at 1:5000 dilution and signal assessed with Super Signal West Pico chemiluminescence substrate (ThermoFisher Scientific, Waltham, MA, USA).

### Preparation of mitochondrial and cytosolic fractions

Mitochondria were isolated by differential centrifugation using the Mitochondria Isolation kit (Abcam, Cambridge, UK) according to the manufacture’s protocol and were solubilized using IP-lysis buffer. Following the isolation of mitochondria, the supernatant representing the cytosol was spun at 22,000×*g* for 30 min and the resultant supernatant was concentrated five-fold in a centrifugal concentrator with a molecular weight cut-off of 10,000 (Merck-Millipore, Burlington, MA).

### Assays of reduced folate pools

Samples were prepared essentially as we described [16]. Fifty milligrams of tissues was homogenized on ice in 1 ml of 50 mM Tris-HCl buffer, pH 7.4 containing 50-mM sodium ascorbate using a Dounce homogenizer and lysed by heating for 3 min in a boiling water bath. In the case of the whole blood, 50 μl of the sample was mixed with 450 μl of the same buffer and treated as above. Lysates were chilled on ice and centrifuged for 5 min at 17,000×*g* at 4 °C. Folate pools were measured in tissue lysates by the ternary complex assay method as we described previously [15, 17]. Folate levels were calculated per milligram of protein measured by Bradford assay or per milliliter of whole blood. Statistical analysis was carried out using GraphPad Prism VIII software. Statistical significance was calculated using Student’s *t* test.

### ATP and NADPH assays

The liver tissue was excised and flash-frozen from mice under isoflurane following IACUC guidelines. ATP and NADPH/NADP^+^ were measured in whole liver lysate or isolated mitochondria using colorimetric ATP and fluorescence NADPH/NADP^+^ kits (Abcam, ab83355 and ab65349), respectively, according to the manufacturer’s protocols. Twenty-five micrograms of the sample were used in the assay. In each case, experiments were carried out in quadruplicates. Statistical analysis was carried out using the GraphPad Prism VIII and JMP software. Statistical significance was calculated using Student’s *t* test.

### Metabolome analysis

Metabolomics was performed using commercial services from Metabolon (Durham, NC). Individual samples (100–200 mg of flash-frozen tissue) were subjected to methanol extraction then split into aliquots for analysis by ultrahigh performance liquid chromatography/mass spectrometry (UHPLC/MS). The global biochemical profiling analysis included four unique arms consisting of reverse phase chromatography-positive ionization methods optimized for hydrophilic compounds (LC/MS Pos Polar) and hydrophobic compounds (LC/MS Pos Lipid), reverse phase chromatography with negative ionization conditions (LC/MS Neg), as well as a HILIC chromatography method coupled to negative ion mode ESI (LC/MS Polar) [18]. All of the methods alternated between full-scan MS and data-dependent MSn scans. The scan range varied slightly between methods but generally covered 70–1000 m/z. Metabolites were identified by automated comparison of the ion features in the experimental samples to a reference library of chemical standard entries that included retention time, molecular weight (m/z), preferred adducts, and in-source fragments as well as associated MS spectra and curated by visual inspection for the quality control using software developed at Metabolon [19]. A detailed description of metabolome-related methodology is provided in Supplementary Methods.

### Statistical analysis

The analysis was carried out essentially as described [5, 20–22]. Two types of statistical analyses were performed: (1) significance tests and (2) classification analysis. Standard statistical analyses were performed in Array Studio software package on log-transformed data. For analyses not standard in Array Studio, the R program (http://cran.r-project.org/) was used. Following log transformation and imputation of missing values, if any, with the minimum observed value for each compound, Welch’s two-sample *t* test was used as a significance test to identify biochemicals that differed significantly (*p* < 0.05) between experimental groups. An estimate of the false discovery rate (*q* value) was calculated to take into account the multiple comparisons that normally occur in metabolomic-based studies. Classification analyses used included principal components analysis (PCA), hierarchical clustering, and OPLS-DA. For the scaled intensity graphics, each biochemical in the original scale (raw area count) was rescaled to set the median across all samples equal to 1.

### NanoString

RNA was extracted using a mRNeasy micro kit (Qiagen, Valencia, CA, Catalog #217084), and the gene expression analysis was performed by the genomics facility of David H. Murdok Research Institute (Kannapolis, NC). Raw intensity values (counts) obtained from the analysis were normalized using counts for six housekeeping genes included in the array.

## Results

### Generation and characterization of *Aldh1l2*^*-/-*^ mice

**Fig. 1:**
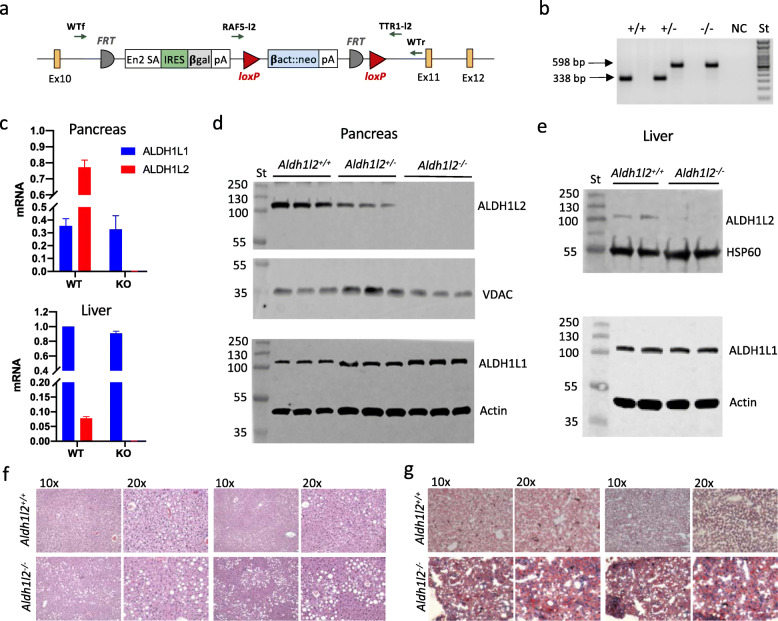
Generation and characterization of *Aldh1l2* knockout mice. **a** Schematic presentation of the *Aldh1l2* gene-trapping vector. Ex, exon; FRT, Flp-recombinase target; loxP, Cre-recombinase site. Primers for mouse genotyping and their approximate target locations are shown (WTf and WTr, primers for the wild type allele; RAF5-l2 and TTR1-l2, primers for the disrupted allele). **b** PCR-based genotyping of the wild type *Aldh1l2* allele (WTf/WTr primer pair) generates a 338 bp fragment, whereas amplification of the disrupted allele (RAF5-l2/TTR1-l2 primer pair) generates a 598-bp fragment. **c** Levels of Aldh1l2 and Aldh1l1 mRNA in the pancreas and liver of wild type and *Aldh1l2* KO mice measured by RT-PCR. **d** Western blot assays of mitochondria (*upper* panels) and cytosol (*lower* panels) from pancreas of *Aldh1l2*^*+/+*^, *Aldh1l2*^*+/−*^, and *Aldh1l2*^*−/−*^ mice (*n* = 3). **e** Western blot assays of mitochondria (*upper* panel) and cytosol (*lower* panel) from livers of *Aldh1l2*^*+/+*^ and *Aldh1l2*^*−/−*^ mice (*n* = 2). *St*, molecular weight standards. **f** H&E and **g** Oil Red O staining of the liver tissue from *Aldh1l2*^*+/+*^ and *Aldh1l2*^*-/-*^ male mice (two mice for each genotype were analyzed)

We generated *Aldh1l2*^*-/-*^ mice using ES cells (clone EPD0514_5_F12 with the targeted non-conditional allele for Aldh1l2) obtained from the NCRR-NIH-supported KOMP Repository (www.komp.org). The non-conditional targeting of *Aldh1l2* was achieved via homologous recombination with the gene-trapping vector depicted in Fig. 1a, whereby the trapping cassette inserted in the intron upstream of exon 11 and resulting in a constitutive null mutation. PCR-based genotyping of the wild type *Aldh1l2* allele generated a 338-bp amplicon, whereas amplification of the mutant allele generated a 598-bp amplicon (Fig. 1b). The successful knockout of the *Aldh1l2* gene was demonstrated by the loss of the ALDH1L2 mRNA and protein in the pancreas, the organ with the highest expression of the enzyme (Fig. 1c, d). Of note, heterozygous *Aldh1l2*^*+/-*^ mice showed a partial decrease in the ALDH1L2 protein levels in the pancreas indicating a gene-dosage effect on the protein expression (Fig. 1d and Supplementary Fig. S1). We have also demonstrated the loss of the enzyme in the liver though its levels in this organ are much lower than in the pancreas (Fig. 1c, d). Analysis of the expression of the cytosolic isoform, ALDH1L1, showed that it was not affected by the loss of the ALDH1L2 protein (Fig. 1c, d).

Both female and male *Aldh1l2*^*-/-*^ mice were viable, fertile, and developed normally with their body weight similar to the weight of wild type littermates at weaning and at the age of 6 months (Supplementary Table S3 and Supplementary Fig. S4). No phenotypic difference between *Aldh1l2*^*-/-*^ and *Aldh1l2*^*+/+*^ mice in terms of growth or food consumption was observed, and knockout mice appeared healthy. Offspring analysis from *Aldh1l2*^*+/-*^ intercrosses (192 pups) showed an average litter size of 6.13 and no deviation from the Mendelian inheritance ratio (slight differences observed for males did not reach significance). Similar litter size (6.1) was observed when mating male or female *Aldh1l2*^*-/-*^ with *Aldh1l2*^*+/+*^ mice, as well as from mating pairs where both sexes were either *Aldh1l2*^*-/-*^ or *Aldh1l2*^*+/+*^ genotype (Supplementary Table S4). Thus, the loss of Aldh1l2 neither affects the mice fertility nor results in embryonic lethality.

### Effect of *Aldh1l2* KO on tissue histology

Gross examination of the brain, lungs, kidney, liver, pancreas, heart, and spleen did not reveal any noticeable differences between genotypes in the organ size, weight, or morphology (Supplementary Figs. S3). H&E staining and histological analysis of the tissues further indicated the lack of differences between the two genotypes (Supplementary Fig. S4). Overall, our study demonstrates that phenotypically, the *Aldh1l2*^*-/-*^ genotype is indistinguishable from the *Aldh1l2*^*+/+*^ genotype in most organs. However, histological changes were noted in the liver of KO male mice. Specifically, the livers of male mice contain increased vacuolar change consistent with the accumulation of lipid in hepatocytes as observed in the fatty livers (Fig. 1f). The vacuolar changes are focused around the central veins with smaller vacuoles closer to the vein and larger vacuoles in the midzonal region. This is an important observation as the blood oxygenation and metabolic capacities of hepatocytes differ based on location within the lobule. Oil Red O staining of liver sections confirmed the high content of lipids in the liver in male mice (Fig. 1g). Overall, these data showed that the male knockouts accumulate more fats in the liver than wild type mice.

### Levels of folate, NADPH, and ATP in *Aldh1l2* KO mice

**Fig. 2:**
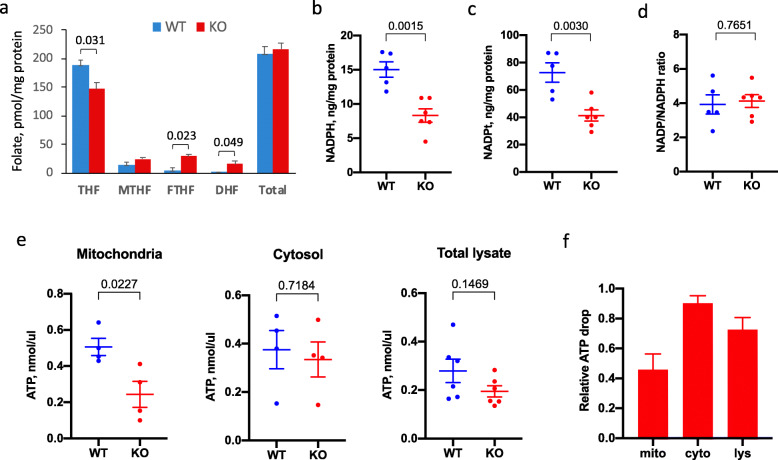
Levels of ALDH1L2-relevant metabolites in the liver of *Aldh1l2*^*+/+*^ and *Aldh1l2*^*−/−*^ male mice. **a** Levels of reduced folate pools (FA, folic acid; THF, tetrahydrofolate; 5-MTHF, 5-methyl-THF; 10-CHO-THF, 10-formyl-THF). For each group, samples from 3 mice were analyzed in quadruplicate. **b**–**d** Levels of NADPH, NADPH plus NADP^+^ (NADPt), and the ratio between oxidized/reduced coenzyme. Five and six mice were analyzed for *Aldh1l2*^*+/+*^ (WT) and *Aldh1l2*^*−/−*^ (KO) groups, respectively; analysis was done in quadruplicate. **e** Levels of ATP in mitochondria, cytosol, and total liver lysates. Number of biological replicates (**b**–**e**) correspond to the number of dots; for each sample, measurements were performed in quadruplicate. **f** Relative changes in ATP between *Aldh1l2*^*+/+*^ and *Aldh1l2*^*-/-*^ mice. Numbers in **a**–**e** show *p* values (statistical analysis was carried out using graph pad Prism VIII software. Data are expressed as mean ± SE. Statistical significance was calculated using Student’s *t* test)

We have evaluated levels of reduced folate pools in the livers of *Aldh1l2*^*-/-*^ and *Aldh1l2*^*+/+*^ male mice. Our folate assay measures 10-formyl-THF, 5-methyl-THF, the combination of THF/5,10-methylene-THF, and the combination of dihydrofolate (DHF) and folic acid (FA) [15, 17]. We observed statistically significant differences between the genotypes in two folate pools, 10-formyl-THF and FA/DHF, with higher levels of these metabolites found in *Aldh1l2*^*-/-*^ mice (Fig. 2a). Of note, the total folate pool was not different between *Aldh1l2*^*-/-*^ and *Aldh1l2*^*+/+*^ mice (Fig. 2a). The changes in 10-formyl-THF agree with the role of ALDH1L2-catalyzed reaction and correspond to the elevation of this folate pool in a patient lacking ALDH1L2 expression due to compound heterozygous mutations [5].

Since ALDH1L2 produces NADPH and was reported as a major contributor to the NADPH pool in mitochondria [3], we measured levels of NADPH and total NADP^+^/NADPH in liver lysates. These experiments showed that levels of NADPH indeed were significantly (40%) decreased in *Aldh1l2*^*-/-*^ compared to *Aldh1l2*^*+/+*^ mice (Fig. 2b and Supplementary Fig. S5). Interestingly, the total NADP^+^/NADPH pool was also decreased to a similar extent so the ratio between the oxidized and reduced forms remained the same (Fig. 2c, d; Supplementary Figs. S6 & S7). We have also observed a strong decrease in ATP in *Aldh1l2*^*-/-*^ mice (Fig. 2e, f; Supplementary Figs. S8, S9 & S10). These changes were more profound in mitochondria with only marginal changes in the cytosol, and moderate changes in the total lysates that reflect the sum of changes in both compartments (Fig. 2e, f).

### Metabolomic analysis of *Aldh1l2* knockout mouse tissues

**Fig. 3:**
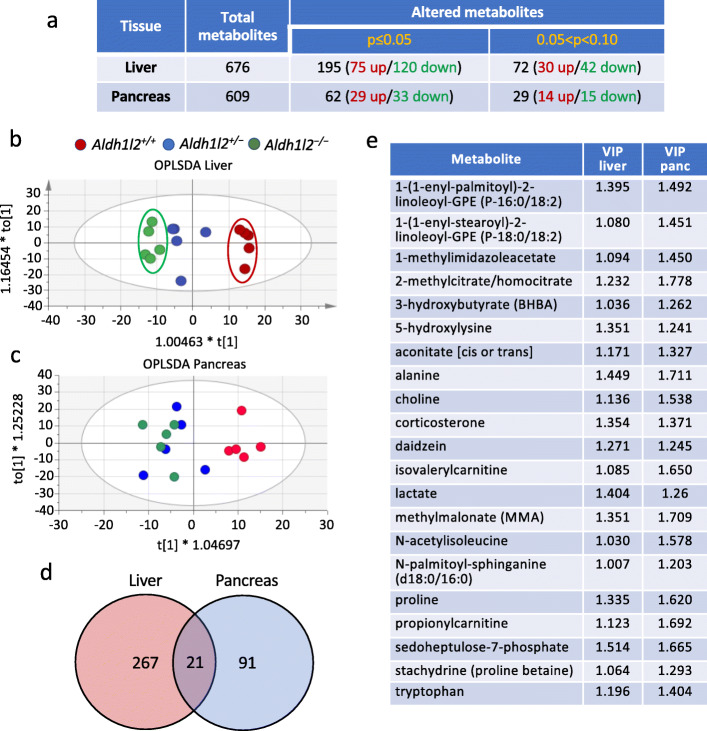
Comparison of the liver and pancreas metabolomic data for *Aldh1l2*^*+/+*^, *Aldh1l2*^*+/−*^, and *Aldh1l2*^*−/−*^ male mice. **a** Summary of metabolome analysis. **b**, **c** OPLS-DA of metabolomic data for the liver and pancreas (three *Aldh1l2* genotypes are included). **d** Venn diagram showing the number of metabolites changed in the liver and pancreas upon ALDH1L2 loss. **e** List of metabolites changed in both the liver and pancreas upon the ALDH1L2 loss

We performed an untargeted metabolomic analysis of the liver and pancreatic tissues isolated from *Aldh1l2*^*+/+*^, *Aldh1l2*^*+/-*^, and *Aldh1l2*^*-/-*^ male mice. In these experiments, lysates were prepared from the whole tissues. In the liver, we observed a strong effect of the KO on the metabolomic profile (Fig. 3a). Specifically, a significant effect of genotype was evident for 267 out of total 676 assigned metabolites (*p* ≤ 0.05 for 195 metabolites and 0.05 < *p* < 0.1 for 72 metabolites) with OPLS-DA showing good segregation between genotypes and the gene-dosage dependence (Fig. 3b). Though no effect of the KO on levels of folate was observed in this analysis, it should be emphasized that this metabolomic approach typically measures a limited number of folate coenzymes (5-MTHF, FA, and dihydrofolate) and only their mono-glutamylated forms; thus, these values do not reflect the actual folate changes, especially if they are not large. In the pancreas, *Aldh1l2* KO resulted in statistically significant changes only for 91 metabolites (*p* ≤ 0.05 for 62 metabolites and 0.05 < *p* < 0.1 for 29 metabolites, Fig. 3a). OPLS-DA showed good separation of pancreatic metabotypes between *Aldh1l2*^*+/+*^ and *Aldh1l2*^*-/-*^ mice, but the metabotype of the heterozygous *Aldh1l2*^*+/-*^ mice was more similar to the metabotype of the knockout mice (Fig. 3c). Twenty-one metabolites that differentiated the study groups were common between the two tissues (Fig. 3d, e).

### Effect of *Aldh1l2* KO on the liver metabolome

**Fig. 4:**
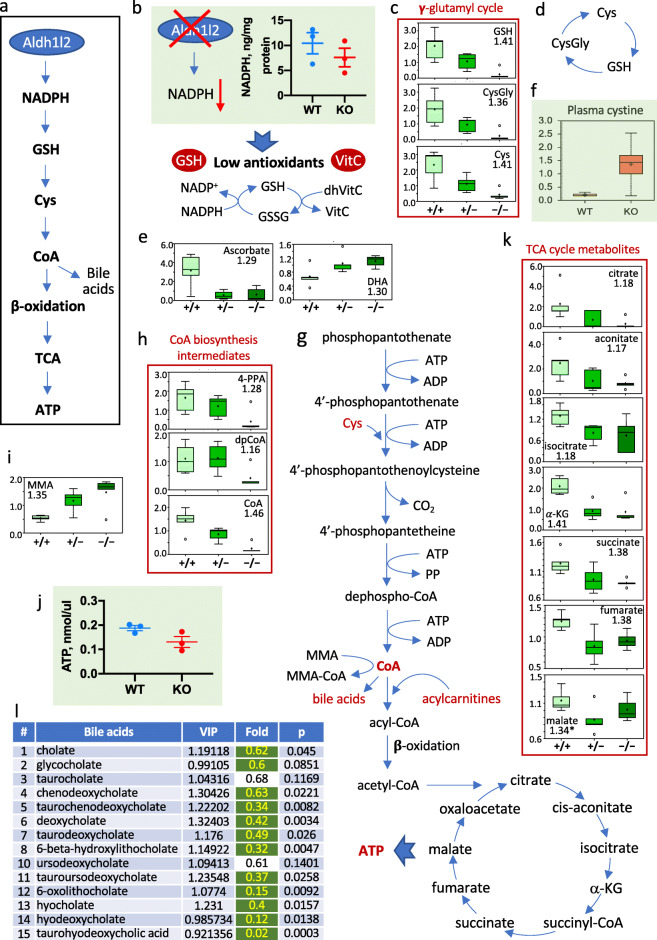
Metabolomic analysis of *Aldh1l2*^*+/+*^, *Aldh1l2*^*+/−*^, and *Aldh1l2*^*−/−*^ male mice links metabolic changes to ALDH1L2 function. **a** Proposed sequence of linked metabolic nodes downstream of ALDH1L2 catalysis. **b** The ALDH1L2 loss causes NADPH decrease; schematic depicts the mechanism of associated decrease in antioxidants, GSH and ascorbate. **c**–**e** Levels of γ-glutamyl cycle metabolites and ascorbate are diminished while levels of dehydroascorbate are increased in *Aldh1l2 KO* mice. **f** Cystine is strongly elevated in plasma of *Aldh1l2 KO* mice. **g** Coenzyme A biosynthesis and pathways downstream of the coenzyme affected by the *Aldh1l2* loss. **h**–**l** Intermediates of coenzyme A biosynthesis, TCA cycle metabolites, bile acids, and ATP are decreased while methylmalonic acid (MMA) is elevated in *Aldh1l2* KO mice

Analysis of metabolomic data in the liver of *Aldh1l2*^*+/+*^, *Aldh1l2*^*+/-*^, and *Aldh1l2*^*-/-*^ mice showed strong changes in numerous metabolites from a variety of pathways (Supplementary Data File 1), which could be a result of the decreased NADPH (Fig. 4a, b). Insufficient production of NADPH in mitochondria is commonly associated with decreased cellular capacity to remove ROS leading to increased oxidative stress [23–25]. Our data indicate that levels of common antioxidants, GSH and cysteine, are drastically decreased (8.3-fold and 5.6-fold, respectively) in *Aldh1l2* KO mice (Fig. 4c). In support of the loss of the reductive potential of the cell, levels of ascorbate (vitamin C) decreased 5-fold while levels of dehydroascorbate (oxidized vitamin C) increased 1.66-fold, dropping the reduced/oxidized vitamin C ratio more than 8-fold (Supplementary Data File 1 and Fig. 4e). GSH also serves as a storage for cysteine, with GSH, cysteinylglycine and cysteine being a part of γ-glutamyl cycle (simplistically illustrated in Fig. 4d) [26]. Three key metabolites of this cycle are strongly decreased in *Aldh1l2* KO mice (Fig. 4c), the finding indicating that the loss of the gene limits cysteine availability and thus decreases the liver’s capacity for cysteine-dependent reactions. Cysteine is directly obtained from the diet or synthesized from methionine via the methionine cycle and transsulfuration pathway [27]. Apparently, the latter pathway does not compensate for the oxidation-linked loss of cysteine in *Aldh1l2*^*-/-*^ mice: the intermediate of the pathway, cystathionine is reduced in KO mice (Supplementary Data File 1). In support of the loss of cysteine due to oxidation, levels of cystine are elevated in plasma of KO mice 5.18-fold, *p* = 0.011 (Fig. 4f and Supplementary Data File 3).

One of the key cysteine-required pathways, the coenzyme A (CoA) biosynthesis [28], is strongly downregulated in Aldh1l2 KO mice (Fig. 4g, h). Three consecutive metabolites within the pathway downstream of the cysteine-required step, including the final product CoA, are significantly decreased in the KO compared to wild type mice (Fig. 4h). Further analysis showed that several CoA-dependent pathways are altered in KO mice. This finding indicates that upon the ALDH1L2 loss, the CoA deficiency has a strong impact on liver metabolism. Specifically, the accumulation of methylmalonic acid (observed in both the liver and pancreas), the dramatic drop in the bile acid levels, and the accumulation of acylcarnitines are in agreement with a low abundance of CoA (Fig. 4g–i and Supplementary Data File 1). One of the most crucial pathways requiring CoA, β-oxidation of fatty acid, is also expected to be downregulated in the KO mice (Fig. 4g). Both the accumulation of acylcarnitines and decreased intermediates of TCA cycle support this mechanism (Supplementary Data File 1 and Fig. 4k). In agreement with a lower capacity of the TCA cycle, the level of ATP in the liver of *Aldh1l2* KO mice was decreased (Fig. 4l).

### Metabolomic analysis of *Aldh1l2* knockout mouse plasma

**Fig. 5:**
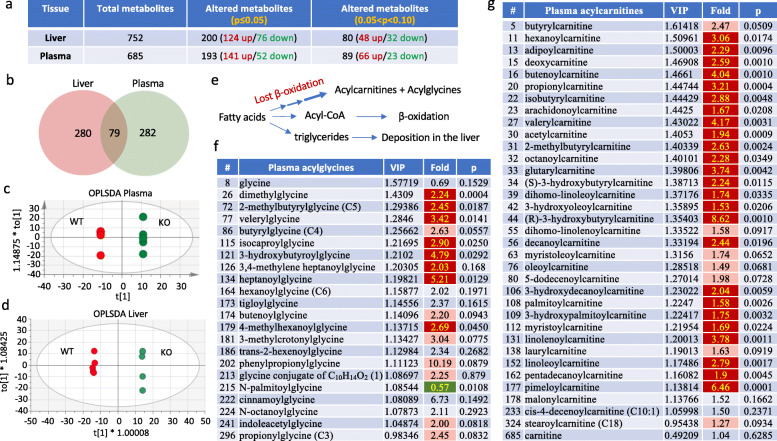
Comparison of the liver and plasma metabolomic data for wild type and *Aldh1l2* KO male mice. **a** Summary of metabolome analysis. **b** Venn diagram showing metabolites changed in liver and plasma upon ALDH1L2 loss. **c**, **d** OPLS-DA of metabolomic data for the plasma and liver of wild type and KO mice. **e** Schematic depicting the fate of fatty acids: in the case of β-oxidation impairment (linked to CoA deficiency in *Aldh1l2* KO mice) fatty acids are conjugated with carnitine and glycine and directed to the blood or converted to triglycerides and deposited in the liver. **f**, **g** Acylcarnitines and acylglycines are elevated in plasma of *Aldh1l2* KO mice

In a separate experiment, we analyzed metabolites in the plasma of *Aldh1l2*^*-/-*^ and *Aldh1l2*^*+/+*^ mice. In parallel with plasma analysis, we also analyzed the liver metabolome. Mouse groups in this experiment were different from the first experiment described above. A total of 685 metabolites were assigned in plasma and 752 in the liver; 282 and 280 metabolites, respectively, showed statistically significant changes (Fig. 5a and Supplementary Data File 3) with 79 perturbed metabolites overlapping between both tissues (Fig. 5b and Supplementary Data File 5). Good segregation between genotypes was evident in both tissues (Fig. 5c, d). We noted a strong increase in numerous acylcarnitines in plasma of *Aldh1l2*^*-/-*^ compared to *Aldh1l2*^*+/+*^ mice (Fig. 5e, g), which is in agreement with the elevation of these metabolites in the liver and pancreas (Supplementary Data File 1). Furthermore, many acylglycine conjugates are elevated in the plasma of *Aldh1l2*^*-/-*^ compared to *Aldh1l2*^*+/+*^ mice (Fig. 5f). Six elevated acylcarnitine conjugates and five acylglycine conjugates are common between the liver and plasma (Supplementary Data File 5). Of note, numerous intermediates of amino acid degradation pathways are markedly elevated in KO mice (Supplementary Data File 4). As well, intermediates of amino acid metabolism are elevated in the liver though to a lesser extent (Supplementary Data File 3). Also, our analysis showed the accumulation of advanced glycation end products N6-carboxymethyllysine and 1-carboxyethylated valine (Supplementary Data Files 3 and 5). Elevation of additional carboxyethylated metabolites, 1-carboxyethylisoleucine, 1-carboxyethyltyrosine, and 1-carboxyethylphenylalanine was observed (Supplementary Data File 3). By analogy, it may be assumed that these are also advanced glycation end-products. However, there is no literature regarding the origin or roles of these metabolites. Overall, plasma metabolome indicated a strong effect of the *Aldh1l2* KO on metabotype that supports our findings from the liver metabolome analysis.

### *Aldh1l2* KO versus *Aldh1l1* KO in the effect on inflammation pathways

One of the goals of this study was to differentiate the function of the ALDH1L2 enzyme from the function of its cytosolic counterpart ALDH1L1. We previously reported that the *Aldh1l1* KO in mice affected genes associated with inflammation [12]. The role of the folate metabolism in inflammation and immunity has been underscored [29, 30]. Metabolomic analysis of *Aldh1l2* KO mice indicates that these mice are likely to experience enhanced oxidative stress. Oxidative stress is highly correlative with inflammation, which is an essential component of the immune response [31]. Though *Aldh1l2* KO (Supplementary Data Files 1 and 3) and *Aldh1l1* KO [12] have strictly different metabotypes, both genotypes were associated with the diminished antioxidant pool in the liver. The effect was especially profound for the *Aldh1l2*^*-/-*^ genotype where both antioxidants, reduced GSH and ascorbate, are strongly decreased (Fig. 4c, e). These findings raised the question of whether the loss of *Aldh1l2* affects the expression of genes associated with inflammation in a manner similar to the *Aldh1l1* KO.

**Fig. 6:**
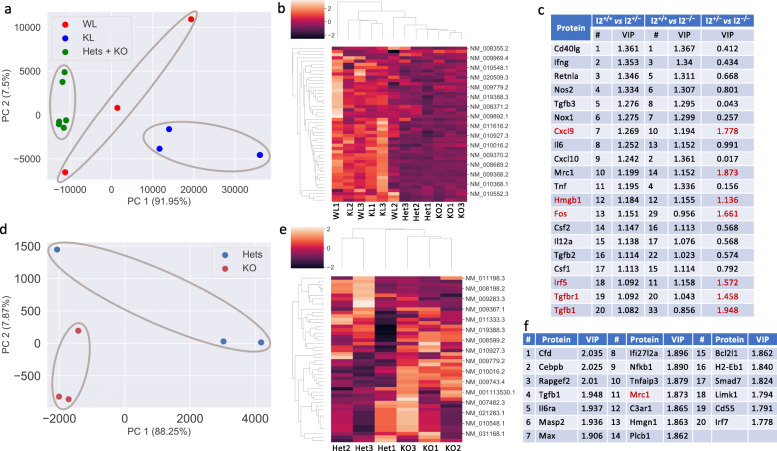
Bioinformatics analysis of NanoString data for livers of *Aldh1l2*^*+/+*^ and *Aldh1l2*^*−/−*^ male mice. PCA was carried out using the *sklearn* package in Python. PLS-DA analysis was carried out using the package *mixOmics* in R. **a** PCA for wild type (WL), *Aldh1l1*^*−/−*^ (KL), *Aldh1l2*^*+/−*^ (Hets), and *Aldh1l2*^*−/−*^ (KO) mice. Raw data were first normalized and then PCA was applied. No scaling was used prior to PCA. PC1 and PC2 explain 91.95% and 7.5% of the total variance of the data, respectively. **b** Heat map representation of the NanoString data for the four genotypes (see Supplementary Fig. S11 for the full-size image). Each protein was standardized so that it has mean 0 and standard deviation 1. **c** Top 20 discriminating proteins for *Aldh1l*^*+/−*^ and *Aldh1l2*^*−/−*^ from wild type mice according to PLS-DA VIP values. Most of these genes do not discriminate between *Aldh1l2*^*+/−*^ and *Aldh1l2*^*−/−*^ genotypes. **d** PCA for *Aldh1l2*^*+/−*^ (Hets) and *Aldh1l2*^*−/−*^ (KO) mice. Raw data were first normalized and then PCA was applied. No scaling was used prior to PCA. PC1 and PC2 explain 88.25% and 7.87% of the total variance of the data, respectively. **e** Heat map representation of the NanoString data for the *Aldh1l*^*+/−*^ and *Aldh1l2*^*−/−*^ genotypes (based on the entire panel of 242 genes; see Supplementary Fig. S12 for the full-size image). Each protein was standardized so that it has mean 0 and standard deviation 1. **f** Top 20 discriminating proteins for *Aldh1l*^*+/−*^ versus *Aldh1l2*^*−/−*^ genotype according to PLS-DA VIP values

To investigate the effect of *Aldh1l2* knockout on gene expression profile, we performed NanoString analysis of mRNAs from the livers of 3-month-old male mice (Supplementary Data File 6) using a commercial inflammation panel and matched it with our previous analysis of Aldh1l1 KO, where a custom panel was used [12]. The present panel includes a total of 242 genes relevant to inflammation and the immune response. The comparison between groups is based on 48 genes overlapping between the two panels (Supplementary Data File 6). PCA showed that *Aldh1l2*^*-/-*^ mice strongly segregate from both WT and *Aldh1l1*^*-/-*^ mice (Fig. 6a). Our analysis indicated that *Aldh1l2* KO and *Aldh1l1* KO groups segregate from each other much stronger than each of them from the WT group, the finding suggesting a vastly different effect of each KO on gene expression in the liver. Based on 48 genes, *Aldh1l2*^*–/–*^ and *Aldh1l2*^*+/–*^ genotypes were close to each other but different from *Aldh1l2*^*+/+*^ and *Aldh1l1*^*–/–*^ genotypes (Fig. 6a, b). To further identify transcripts that contribute most to the discrimination between the wild type and heterozygous or homozygous *Aldh1l2* knockout mice, we performed a multivariate analysis applying a supervised machine learning method to the standardized data. Unlike the unsupervised PCA, applied Partial Least Squares-Discriminant Analysis (PLS-DA) takes advantage of the known sample type information and can quantify the relative contribution of each protein to the separation of the genotypes by using the VIP value [32]. Figure 6c shows the top 20 discriminating proteins based on the PLS-DA analysis. Among the top 20 targets differentiating either the *Aldh1l2*^*–/–*^ or *Aldh1l2*^*+/–*^ genotype from the wild type genotype, 17 genes overlap (Fig. 6c) suggesting a strong effect of the single allele loss. Our study further demonstrated good segregation between *Aldh1l2*^*–/–*^ and *Aldh1l2*^*+/–*^ groups when a greater number of genes are included in the analysis (Fig. 6d, e). Noticeably, genes differentiating *Aldh1l2*^*–/–*^ and *Aldh1l2*^*+/–*^ genotypes do not overlap with genes differentiating both genotypes from the wild type (Fig. 6f). Overall, these data suggest that ALDH1L2 protein expression is important for the control of inflammation in the liver.

Several targets differentiating the *Aldh1l2*^*-/-*^/*Aldh1l2*^*+/-*^ group from WT/Aldh1l1 KO group, but not *Aldh1l2*^*-/-*^ group from *Aldh1l2*^*+/-*^ group are linked to metabolomic changes associated with complete or partial Aldh1l2 loss. Thus, changes in intermediates of the urea cycle and arginine metabolism would fit to alteration of expression of inducible nitric oxide synthase 2 (*Nos2*) and arginase (*Arg1*) while the effect on NADPH and oxidative stress would explain changes in the expression of the former gene as well as changes in NADPH oxidase 1 (*Nox1*). Of note, strong accumulation of 4-guanidinobutanoate in the liver and plasma of *Aldh1l2* KO mice is also in agreement with arginase deficiency [33].

## Discussion

Two closely related enzymes in the cell, cytosolic ALDH1L1 and mitochondrial ALDH1L2, catalyze the same biochemical reaction, the conversion of 10-formyl-THF to THF and CO_2_ [34]. This reaction simultaneously produces NADPH from NADP^+^. It is not clear whether these enzymes have overlapping metabolic functions or whether each reaction serves different purposes. ALDH1L1 is suggested as a regulator of the folate metabolism, the function closely associated with the regulation of cellular proliferation [10, 11, 13]. In agreement with such function, ALDH1L1 is strongly and ubiquitously downregulated in many human cancers through the promoter methylation [35]. The role of ALDH1L2 is not well documented. Of note, ALDH1L2 expression in the liver, the main organ of folate metabolism, is very low compared to the expression of ALDH1L1, which is one of the most abundant proteins in liver cytosol [8]. On the other hand, ALDH1L2 is highly expressed in the pancreas where its levels are even higher than levels of ALDH1L1 [2], the finding which was confirmed in the present study. In contrast to the cytosolic enzyme, ALDH1L2 is expressed in cancer cell lines and is upregulated in colorectal tumor tissues [2, 36]. Furthermore, it may play a role in promoting cancer metastasis as it was shown in melanomas [4]. It was reported that in cultured cancer cells ALDH1L2 is a primary contributor to mitochondrial NADPH [3] and thus may be involved in the oxidative stress response mechanism. Also, several studies have indicated that ALDH1L2 could be a stress-response target for certain drugs (reviewed in [34]).

Our study demonstrates that despite the fact that ALDH1L2 is expressed in the liver at a low level, its knockout in mice produces a strong effect on liver metabotype (Supplementary Data File 1). Notably, the *Aldh1l2* KO induced changes in the liver folate pool similar to those produced by *Aldh1l1* KO [12] with a very strong effect on 10-formyl-THF, which was highly elevated in both *Aldh1l2*^*–/–*^ and *Aldh1l1*^*–/–*^ genotypes. Previously, such effect of the ALDH1L2 loss was observed in fibroblast cell culture obtained from a patient who lost the enzyme expression as a result of deleterious compound mutations in *ALDH1L2* [5]. *Aldh1l2*^*-/-*^ mice also have highly elevated dihydrofolate in the liver, the phenomenon which may be associated with the shortage of NADPH required for the reduction of dihydrofolate to THF. The loss of *Aldh1l2* had a very strong effect on lipids, which we also attribute to the insufficient NADPH generation. The sequence of events underlying this link would be as follows: low NADPH limits the reduction of GSSG to GSH; this in turn decreases the ability of the cell to maintain sufficient levels of cysteine (by reducing cystine) and leads to the low availability of cysteine for biochemical reactions including CoA biosynthesis (Fig. 4g). In fact, our metabolomic data support this model. As an ultimate outcome, insufficient CoA would impair β-oxidation and have a profound effect on fatty acid metabolism.

Overall metabolomic changes in mice due to the *Aldh1l2* loss were very different from the changes observed upon the knockout of the cytosolic isoform ALDH1L1 [12]. As we have recently reported, ALDH1L1 is important for the regulation of glycine metabolism in the liver with levels of glycine and numerous glycine conjugates being strongly decreased in the liver of Aldh1l1 KO mice [12]. In contrast to these findings, acylglycine conjugates were uniformly elevated in *Aldh1l2*^*-/-*^ mice without changes in levels of glycine itself. The mechanism underlying such differences is the decreased glycine production upon the *Aldh1l1* loss causing low glycine availability for biochemical reactions versus enhanced glycine conjugation with fatty acids upon the *Aldh1l2* loss. We suggest that the latter process is the result of impaired β-oxidation in *Aldh1l2*^*-/-*^ mice. Indeed, the accumulation of non-oxidized fatty acids associated with disorders of β-oxidation promotes their conjugation with glycine and carnitine [37]. Likewise, the strong elevation of acylcarnitines as well as acylglycines in *Aldh1l2*^*-/-*^ mice is the most noticeable metabolite changes in the plasma. Of note, the elevation of acylcarnitines with different acyl chain length takes place in KO mice. Interestingly, we have previously observed similar changes in levels of acylcarnitines in skin fibroblasts derived from a patient with lost ALDH1L2 expression [5]. Our findings are also in agreement with the fact that the liver is the major contributor to systemic short-chain acylcarnitines [38] with plasma acylcarnitines reflecting mitochondrial β-oxidation in the liver in a mouse model [39]. Since the pathogenesis of the liver fat accumulation includes impaired fatty acid oxidation as one of the possible causes, the accumulation of lipids in the liver of *Aldh1l2* KO mice fits well with the proposed mechanism linking the ALDH1L2 loss to impaired β-oxidation [40]. For example, altered hepatic fatty acid metabolism in nonalcoholic fatty liver disease commonly leads to the accumulation of triglycerides within hepatocytes [41].

Our analysis indicates that the cause of impaired β-oxidation in the liver of ALDH1L2-deficient mice is insufficient CoA biosynthesis, the phenomenon we link to the low availability of cysteine. In support of the CoA-linked mechanism, there are indications in the literature that acylcarnitine concentrations and the relative carnitine pool composition reflect the intra-mitochondrial acyl-CoA to free CoA ratio with a high sensitivity [42]. We propose that the shortage of cysteine in our model is not due to its low synthesis or low dietary availability but is rather associated with oxidative stress due to decreased NADPH levels and eventually with impaired metabolism of GSH, which led to its deficiency. Within the cell, mitochondrial GSH is the main defense against physiological oxidative stress generated by cellular respiration [26, 43, 44]. Altered GSH homeostasis in Aldh1l2 KO mice is further supported by the shift in levels of ascorbate/dehydroascorbate towards a more oxidative state. Indeed, GSH and ascorbate are the most abundant reducing agents in the cell with GSH being capable of non-enzymatic reduction of dehydroascorbate to ascorbate as well as participating in enzymatic dehydroascorbate reduction [45]. GSH fulfills several other essential functions in the cell including storage and transfer of cysteine [26, 43]. GSH is present in all mammalian cells but may be especially important for organs such as the liver with intensive aerobic respiration and exposure to exogenous toxins. In fact, though GSH homeostasis of the organism is a highly complex process, it is predominantly regulated by the liver [46]. Elevated levels of ophthalmate and oxoproline in plasma of *Aldh1l2* KO mice are as well indicative of impaired glutathione biosynthesis and hepatic glutathione depletion [47, 48].

The present study demonstrates that the ALDH1L2 function is important to support systemic metabolic homeostasis and thus may have a more far-reaching importance with regard to human diseases. For example, elevated plasma levels of C3 and C5 acylcarnitines, the phenomenon linked to the ALDH1L2 loss, could be associated with obesity, metabolic syndrome, insulin resistance, and type 2 diabetes [49, 50]. Such an increase was shown to reflect decreased mitochondrial function or mitochondrial impairment [51, 52]. Also, plasma concentrations of butyrylcarnitine and methylbutyrylcarnitine were higher in NASH [53]. Additionally, plasma levels of acylcarnitines are a part of the signature separating health and disease for metabolic syndrome in Dutch middle-aged individuals [54]. The role of ALDH1L2 in the regulation of mitochondrial function, β-oxidation, and acylcarnitine levels suggests that the enzyme could be especially relevant to diabetes. In fact, increased plasma acylcarnitines were identified as predictive markers of mitochondrial disfunction in type 2 diabetes [55] which could be associated with incomplete β-oxidation of fatty acids [56]. Mechanistically, mitochondrial overload might generate by-products, which would affect the insulin signaling pathway, thus leading to insulin resistance [52]. In agreement with this hypothesis, mitochondrial β-oxidation is altered in patients with type 2 diabetes [57]. These alterations are likely caused by nutrient overload and are especially pronounced in obese patients. Accordingly, patients with type 2 diabetes show elevation of short chain acylcarnitines (C2 and C4) [57]. Elevated plasma levels of short-chain acylcarnitines are also associated with the increased risk of gestational diabetes mellitus [58] and were among predictive markers for the development of diabetic nephropathy [59]. Though future studies should shed light on the role of ALDH1L2 in diabetes, changes of several metabolites in *Aldh1l2 KO* mice recorded in the present work indicate the link between *Aldh1l2* and diabetes. Such link is supported by alterations of levels of end-products of protein glycation [60], the glucose marker 1,5-anhydroglucitol [61], and 3-methylhistidine [62] in *Aldh1l2* KO mice.

Metabolomic analysis of *Aldh1l2* KO mice indicates that these mice are likely to experience enhanced oxidative stress. Oxidative stress is highly correlative with inflammation, which is an essential component of the immune response [31]. Furthermore, we have previously shown that ALDH1L2 function is important for the maintenance of mitochondrial integrity [5]. Mitochondrial dysfunction linked to excessive ROS production was increasingly implicated in inflammatory responses and associated diseases (reviewed in [63]). In this context, ALDH1L2 is likely to be involved in inflammatory responses as well. Our analysis of the gene expression relevant to inflammation and immunity indicates that the ALDH1L2 loss indeed affected the expression pattern of these genes. Notably, *Aldh1l2* KO mice are very different from both wild type mice and *Aldh1l1* KO mice in regard to the expression profiles of inflammation-related proteins, further suggesting non-overlapping functions of the cytosolic and mitochondrial 10-formyl-THF metabolizing reactions. Thus, PCA shows that *Aldh1l2*^*-/-*^ and *Aldh1l2*^*+/-*^ mouse groups segregated much better from both wild type and Aldh1l1 KO mice than from each other. However, when compared separately based on a larger number of genes, strong differentiation between *Aldh1l2*^*-/-*^ and *Aldh1l2*^*+/-*^ groups is evident. The top target differentiating the *Aldh1l2* KO group from the WT/*Aldh1l1* KO group is interferon gamma, which would be in agreement with recent findings that interferon signaling is responsive to folate status in murine mammary cancer cells [29]. Interestingly, several targets differentiating the *Aldh1l2*^*-/-*^/*Aldh1l2*^*+/-*^ group from WT/Aldh1l1 KO group but not *Aldh1l2*^*-/-*^ group from *Aldh1l2*^*+/-*^ group might be linked to metabolomic changes associated with complete or partial loss of the protein. Thus, changes in intermediates of the urea cycle and arginine metabolism would fit to alteration of expression of inducible nitric oxide synthase 2 (*Nos2*) and arginase (*Arg1*) while the effect on NADPH and oxidative stress would explain changes in the expression of the former gene as well as changes in NADPH oxidase 1 (*Nox1*). Of note, strong accumulation of 4-guanidinobutanoate in the liver and plasma of Aldh1l2 KO mice is also in agreement with arginase deficiency [33].

## Conclusions

We recently reported that ALDH1L2 maintains mitochondrial integrity in skin fibroblasts and the loss of the enzyme leads to a neuro-cutaneous disease [5]. The present study further underscores this mitochondrial enzyme as an important regulator of fatty acid β-oxidation in the liver. Numerous studies demonstrated that folate and lipid pathways interact in the liver, which is not surprising since the liver is the main organ for both folate and lipid metabolism [64, 65]. This interaction, however, is mainly viewed as the influence through folate-related methylation associated with the availability of methyl group donors [66]. Our study points toward an additional mechanism by which folate metabolism can affect lipid biogenesis, through the oxidative stress-linked regulation of β-oxidation and highlighted the unique role of ALDH1L2 enzyme in this process. Interestingly, numerous metabolic changes in *Aldh1l2* KO mice reflect those determined in skin fibroblasts from the patient with SLS-like symptoms linked to the loss of the gene [5]. This includes decreased NADPH and ATP in the liver and elevated acylcarnitines in the liver and plasma of the KO mice. While inherited defects in complex lipid synthesis, including Sjogren-Larsson syndrome, is likely to affect all organs/tissues [67], specific liver pathology was not reported for SLS patients. However, the *Aldh3a2* gene linked to the SLS etiology is most highly expressed in the liver of mice [68]. Our study also indicates that the function of mitochondrial ALDH1L2 does not overlap with the function of the cytosolic isozyme ALDH1L1, and the cytosolic homolog does not compensate for the loss of the mitochondrial enzyme. Generation of double KO mice lacking both ALDH1L1 and ALDH1L2 proteins will provide further insight into the functional interaction of the two pathways.

## Supplementary Information

**Additional file 1.**

**Additional file 2.**

**Additional file 3.**

**Additional file 4.**

**Additional file 5.**

**Additional file 6.**

**Additional file 7.**

**Additional file 8.**

## Data Availability

The datasets supporting the conclusions of this article are included within the article and its additional files.

## Abbreviations

ALDH1L2: Aldehyde dehydrogenase 1 family member L2
SLS: Sjogren-Larsson Syndrome
THF: Tetrahydrofolate
DHF: Dihydrofolate
FA: Folic acid
CoA: Coenzyme A
UHPLC: Ultrahigh performance liquid chromatography
PCA: Principal components analysis
OPLSDA: Orthogonal projections to latent structures discriminant analysis
KO: Knockout
WT: Wildtype
MS: Mass spectrometry
PLS-DA: Partial least square-discriminant analysis

## References

[1] Tibbetts AS, Appling DR (2010). Compartmentalization of mammalian folate-mediated one-carbon metabolism. Annu Rev Nutr.

[2] Krupenko NI, Dubard ME, Strickland KC, Moxley KM, Oleinik NV, Krupenko SA (2010). ALDH1L2 is the mitochondrial homolog of 10-formyltetrahydrofolate dehydrogenase. J Biol Chem.

[3] Fan J, Ye J, Kamphorst JJ, Shlomi T, Thompson CB, Rabinowitz JD (2014). Quantitative flux analysis reveals folate-dependent NADPH production. Nature.

[4] Piskounova E, Agathocleous M, Murphy MM, Hu Z, Huddlestun SE, Zhao Z, Leitch AM, Johnson TM, DeBerardinis RJ, Morrison SJ (2015). Oxidative stress inhibits distant metastasis by human melanoma cells. Nature.

[5] Sarret C, Ashkavand Z, Paules E, Dorboz I, Pediaditakis P, Sumner S, Eymard-Pierre E, Francannet C, Krupenko NI, Boespflug-Tanguy O (2019). Deleterious mutations in ALDH1L2 suggest a novel cause for neuro-ichthyotic syndrome. NPJ Genom Med.

[6] Rizzo WB, Jenkens SM, Boucher P (2012). Recognition and diagnosis of neuro-ichthyotic syndromes. Semin Neurol.

[7] Weustenfeld M, Eidelpes R, Schmuth M, Rizzo WB, Zschocke J, Keller MA (2019). Genotype and phenotype variability in Sjogren-Larsson syndrome. Hum Mutat.

[8] Krupenko SA (2009). FDH: an aldehyde dehydrogenase fusion enzyme in folate metabolism. Chem Biol Interact.

[9] Krupenko NI, Holmes RS, Tsybovsky Y, Krupenko SA (2015). Aldehyde dehydrogenase homologous folate enzymes: evolutionary switch between cytoplasmic and mitochondrial localization. Chem Biol Interact.

[10] Krupenko SA, Oleinik NV (2002). 10-formyltetrahydrofolate dehydrogenase, one of the major folate enzymes, is down-regulated in tumor tissues and possesses suppressor effects on cancer cells. Cell Growth Differ.

[11] Khan QA, Pediaditakis P, Malakhau Y, Esmaeilniakooshkghazi A, Ashkavand Z, Sereda V, Krupenko NI, Krupenko SA (2018). CHIP E3 ligase mediates proteasomal degradation of the proliferation regulatory protein ALDH1L1 during the transition of NIH3T3 fibroblasts from G0/G1 to S-phase. PLoS One.

[12] Krupenko NI, Sharma J, Pediaditakis P, Fekry B, Helke KL, Du X, Sumner S, Krupenko SA (2019). Cytosolic 10-formyltetrahydrofolate dehydrogenase regulates glycine metabolism in mouse liver. Sci Rep.

[13] Krupenko SA, Krupenko NI (2019). Loss of ALDH1L1 folate enzyme confers a selective metabolic advantage for tumor progression. Chem Biol Interact.

[14] Anderson DD, Stover PJ (2009). SHMT1 and SHMT2 are functionally redundant in nuclear de novo thymidylate biosynthesis. PLoS One.

[15] Oleinik NV, Krupenko NI, Reuland SN, Krupenko SA (2006). Leucovorin-induced resistance against FDH growth suppressor effects occurs through DHFR up-regulation. Biochem Pharmacol.

[16] Oleinik NV, Helke KL, Kistner-Griffin E, Krupenko NI, Krupenko SA (2014). Rho GTPases RhoA and Rac1 mediate effects of dietary folate on metastatic potential of A549 cancer cells through the control of cofilin phosphorylation. J Biol Chem.

[17] Hoeferlin LA, Oleinik NV, Krupenko NI, Krupenko SA (2011). Activation of p21-dependent G1/G2 arrest in the absence of DNA damage as an antiapoptotic response to metabolic stress. Genes Cancer.

[18] Evans AM, Bridgewater BR, Liu Q, Mitchell MW, Robinson RJ, Dai H, Stewart SJ, DeHaven CD, Miller LAD. High resolution mass spectrometry improves data quantity and quality as compared to unit mass resolution mass spectrometry in highthroughput profiling metabolomics. Matabolomics. 2014;4(2):1-7.

[19] Dehaven CD, Evans AM, Dai H, Lawton KA (2010). Organization of GC/MS and LC/MS metabolomics data into chemical libraries. J Cheminform.

[20] Rafikova O, Meadows ML, Kinchen JM, Mohney RP, Maltepe E, Desai AA, Yuan JX, Garcia JG, Fineman JR, Rafikov R (2016). Metabolic changes precede the development of pulmonary hypertension in the monocrotaline exposed rat lung. PLoS One.

[21] Brown MV, McDunn JE, Gunst PR, Smith EM, Milburn MV, Troyer DA, Lawton KA (2012). Cancer detection and biopsy classification using concurrent histopathological and metabolomic analysis of core biopsies. Genome Med.

[22] Worley B, Powers R (2013). Multivariate analysis in metabolomics. Curr Metabolomics.

[23] Starkov AA (2008). The role of mitochondria in reactive oxygen species metabolism and signaling. Ann N Y Acad Sci.

[24] Nickel A, Kohlhaas M, Maack C (2014). Mitochondrial reactive oxygen species production and elimination. J Mol Cell Cardiol.

[25] Venditti P, Di Stefano L, Di Meo S (2013). Mitochondrial metabolism of reactive oxygen species. Mitochondrion.

[26] Lu SC (2009). Regulation of glutathione synthesis. Mol Aspects Med.

[27] Finkelstein JD (1998). The metabolism of homocysteine: pathways and regulation. Eur J Pediatr.

[28] Leonardi R, Zhang YM, Rock CO, Jackowski S (2005). Coenzyme A: back in action. Prog Lipid Res.

[29] Kok DE, O'Flanagan CH, Coleman MF, Ashkavand Z, Hursting SD, Krupenko SA. Effects of folic acid withdrawal on transcriptomic profiles in murine triple-negative breast cancer cell lines. Biochimie. 2020. 10.1016/j.biochi.2020.04.005 PMC7858693 32304770

[30] Kolb AF, Petrie L (2013). Folate deficiency enhances the inflammatory response of macrophages. Mol Immunol.

[31] Li S, Hong M, Tan HY, Wang N, Feng Y (2016). Insights into the role and interdependence of oxidative stress and inflammation in liver diseases. Oxid Med Cell Longev.

[32] Lee LC, Liong CY, Jemain AA (2018). Partial least squares-discriminant analysis (PLS-DA) for classification of high-dimensional (HD) data: a review of contemporary practice strategies and knowledge gaps. Analyst.

[33] Wang SY, Wang Y, Jin XW, Zhang Y, Chen JS, Ma WW, Wu YH, Wang DC (2015). A urinary metabolomics study of rats after the exposure to acrylamide by ultra performance liquid chromatography coupled with quadrupole time-of-flight tandem mass spectrometry. Mol Biosyst.

[34] Krupenko SA, Krupenko NI (2018). ALDH1L1 and ALDH1L2 folate regulatory enzymes in cancer. Adv Exp Med Biol.

[35] Oleinik NV, Krupenko NI, Krupenko SA (2011). Epigenetic silencing of ALDH1L1, a metabolic regulator of cellular proliferation, in cancers. Genes and Cancer.

[36] Miyo M, Konno M, Colvin H, Nishida N, Koseki J, Kawamoto K, Tsunekuni K, Nishimura J, Hata T, Takemasa I, et al. The importance of mitochondrial folate enzymes in human colorectal cancer. Oncol Rep. 2016;37(1):417-25. 10.3892/or.2016.5264 27878282

[37] Adeva-Andany MM, Carneiro-Freire N, Seco-Filgueira M, Fernandez-Fernandez C, Mourino-Bayolo D (2019). Mitochondrial beta-oxidation of saturated fatty acids in humans. Mitochondrion.

[38] Xu G, Hansen JS, Zhao XJ, Chen S, Hoene M, Wang XL, Clemmesen JO, Secher NH, Haring HU, Pedersen BK (2016). Liver and muscle contribute differently to the plasma acylcarnitine pool during fasting and exercise in humans. J Clin Endocrinol Metab.

[39] Bjorndal B, Alteras EK, Lindquist C, Svardal A, Skorve J, Berge RK (2018). Associations between fatty acid oxidation, hepatic mitochondrial function, and plasma acylcarnitine levels in mice. Nutr Metab (Lond).

[40] Bosy-Westphal A, Braun W, Albrecht V, Muller MJ (2019). Determinants of ectopic liver fat in metabolic disease. Eur J Clin Nutr.

[41] Alves-Bezerra M, Cohen DE (2017). Triglyceride metabolism in the liver. Compr Physiol.

[42] Reuter SE, Evans AM (2012). Carnitine and acylcarnitines: pharmacokinetic, pharmacological and clinical aspects. Clin Pharmacokinet.

[43] Lu SC (1999). Regulation of hepatic glutathione synthesis: current concepts and controversies. FASEB J.

[44] Ribas V, Garcia-Ruiz C, Fernandez-Checa JC (2014). Glutathione and mitochondria. Front Pharmacol.

[45] Linster CL, Van Schaftingen E (2007). Vitamin C. Biosynthesis, recycling and degradation in mammals. FEBS J.

[46] Kretzschmar M (1996). Regulation of hepatic glutathione metabolism and its role in hepatotoxicity. Exp Toxicol Pathol.

[47] Dello SA, Neis EP, de Jong MC, van Eijk HM, Kicken CH, Olde Damink SW, Dejong CH (2013). Systematic review of ophthalmate as a novel biomarker of hepatic glutathione depletion. Clin Nutr.

[48] Geenen S, Yates JW, Kenna JG, Bois FY, Wilson ID, Westerhoff HV (2013). Multiscale modelling approach combining a kinetic model of glutathione metabolism with PBPK models of paracetamol and the potential glutathione-depletion biomarkers ophthalmic acid and 5-oxoproline in humans and rats. Integr Biol (Camb).

[49] Rousseau M, Guenard F, Garneau V, Allam-Ndoul B, Lemieux S, Perusse L, Vohl MC. Associations between dietary protein sources, plasma BCAA and short-chain acylcarnitine levels in adults. Nutrients. 2019;11(1):1-16. 10.3390/nu11010173 PMC6356602 30650556

[50] Mihalik SJ, Goodpaster BH, Kelley DE, Chace DH, Vockley J, Toledo FG, DeLany JP (2010). Increased levels of plasma acylcarnitines in obesity and type 2 diabetes and identification of a marker of glucolipotoxicity. Obesity (Silver Spring).

[51] Johansson PI, Nakahira K, Rogers AJ, McGeachie MJ, Baron RM, Fredenburgh LE, Harrington J, Choi AMK, Christopher KB (2018). Plasma mitochondrial DNA and metabolomic alterations in severe critical illness. Crit Care.

[52] Rossi A, Ruoppolo M, Formisano P, Villani G, Albano L, Gallo G, Crisci D, Moccia A, Parenti G, Strisciuglio P (2018). Insulin-resistance in glycogen storage disease type Ia: linking carbohydrates and mitochondria?. J Inherit Metab Dis.

[53] Kalhan SC, Guo L, Edmison J, Dasarathy S, McCullough AJ, Hanson RW, Milburn M (2011). Plasma metabolomic profile in nonalcoholic fatty liver disease. Metabolism.

[54] Surowiec I, Noordam R, Bennett K, Beekman M, Slagboom PE, Lundstedt T, van Heemst D (2019). Metabolomic and lipidomic assessment of the metabolic syndrome in Dutch middle-aged individuals reveals novel biological signatures separating health and disease. Metabolomics.

[55] Abu Bakar MH, Sarmidi MR (2017). Association of cultured myotubes and fasting plasma metabolite profiles with mitochondrial dysfunction in type 2 diabetes subjects. Mol Biosyst.

[56] Adams SH, Hoppel CL, Lok KH, Zhao L, Wong SW, Minkler PE, Hwang DH, Newman JW, Garvey WT (2009). Plasma acylcarnitine profiles suggest incomplete long-chain fatty acid beta-oxidation and altered tricarboxylic acid cycle activity in type 2 diabetic African-American women. J Nutr.

[57] Villarreal-Perez JZ, Villarreal-Martinez JZ, Lavalle-Gonzalez FJ, Torres-Sepulveda Mdel R, Ruiz-Herrera C, Cerda-Flores RM, Castillo-Garcia ER, Rodriguez-Sanchez IP, Martinez de Villarreal LE (2014). Plasma and urine metabolic profiles are reflective of altered beta-oxidation in non-diabetic obese subjects and patients with type 2 diabetes mellitus. Diabetol Metab Syndr.

[58] Roy C, Tremblay PY, Anassour-Laouan-Sidi E, Lucas M, Forest JC, Giguere Y, Ayotte P (2018). Risk of gestational diabetes mellitus in relation to plasma concentrations of amino acids and acylcarnitines: a nested case-control study. Diabetes Res Clin Pract.

[59] Pena MJ, Lambers Heerspink HJ, Hellemons ME, Friedrich T, Dallmann G, Lajer M, Bakker SJ, Gansevoort RT, Rossing P, de Zeeuw D (2014). Urine and plasma metabolites predict the development of diabetic nephropathy in individuals with type 2 diabetes mellitus. Diabet Med.

[60] Jagadeeshaprasad MG, Batkulwar KB, Meshram NN, Tiwari S, Korwar AM, Unnikrishnan AG, Kulkarni MJ (2016). Targeted quantification of N-1-(carboxymethyl) valine and N-1-(carboxyethyl) valine peptides of beta-hemoglobin for better diagnostics in diabetes. Clin Proteomics.

[61] Kim WJ, Park CY (2013). 1,5-Anhydroglucitol in diabetes mellitus. Endocrine.

[62] Zhou Y, Qiu L, Xiao Q, Wang Y, Meng X, Xu R, Wang S, Na R (2013). Obesity and diabetes related plasma amino acid alterations. Clin Biochem.

[63] West AP, Shadel GS (2017). Mitochondrial DNA in innate immune responses and inflammatory pathology. Nat Rev Immunol.

[64] Nguyen P, Leray V, Diez M, Serisier S, Le Bloc'h J, Siliart B, Dumon H (2008). Liver lipid metabolism. J Anim Physiol Anim Nutr (Berl).

[65] Sid V, Siow YL (2017). O K: Role of folate in nonalcoholic fatty liver disease. Can J Physiol Pharmacol.

[66] da Silva RP, Kelly KB, Al Rajabi A, Jacobs RL (2014). Novel insights on interactions between folate and lipid metabolism. Biofactors.

[67] Garcia-Cazorla A, Mochel F, Lamari F, Saudubray JM (2015). The clinical spectrum of inherited diseases involved in the synthesis and remodeling of complex lipids. A tentative overview. J Inherit Metab Dis.

[68] Naganuma T, Takagi S, Kanetake T, Kitamura T, Hattori S, Miyakawa T, Sassa T, Kihara A (2016). Disruption of the Sjogren-Larsson syndrome gene Aldh3a2 in mice increases keratinocyte growth and retards skin barrier recovery. J Biol Chem.

