# Supplementary material for: Aldh1l2 knockout mouse metabolomics links the loss of the mitochondrial folate enzyme to deregulation of a lipid metabolism observed in rare human disorder
Source: Hum Genomics. 2020 Nov 9;14:41. doi: 10.1186/s40246-020-00291-3 (PMC7654619; doi:10.1186/s40246-020-00291-3)
Supplement: Supplementary file 1 — Additional file 1. [file 40246_2020_291_MOESM1_ESM.pdf]

SUPPLEMENT (Krupenko et al)

1. Supplementary figures S1-S12

2. Supplementary Tables S1-S4

Supplementary Data Files 1-6 (separate Excel files)

Supplementary Data Files 1: liver and pancreas metabolomic data (three genotypes)

Supplementary Data File 2: VIP values (OPLSDA) for metabolomic data, liver and pancreas (three genotypes)

Supplementary Data Files 3: liver and plasma metabolomic data (wild type and Aldh1l2 KO, second experiment)

Supplementary Data Files 4: VIP values (OPLSDA) for metabolomic data, liver and plasma (wild type and Aldh1l2 KO, second experiment)

Supplementary Data File 5: list of metabolites overlapping between liver and plasma

Supplementary Data File 6: NanoString data and VIP values (OPLSDA analysis)

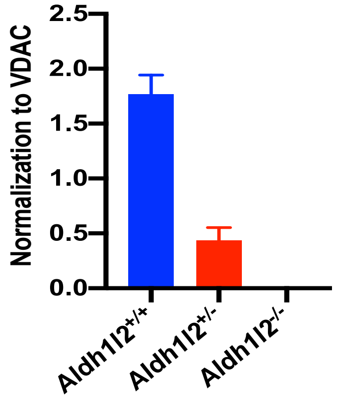

Supplementary Fig. S1. Intensity of ALDH1L2 bands (mean ± SE) relative to VDAC (from Fig. 1d; calculated using Image J).

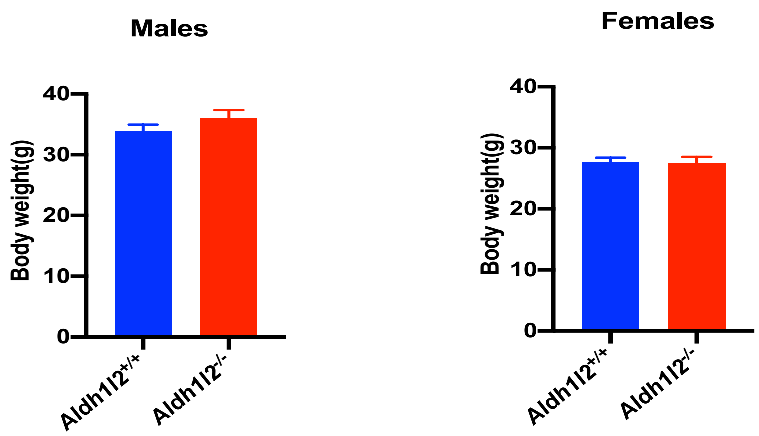

Supplementary Fig. S2. Body weights of *Aldh1l2*$^{+/+}$ and *Aldh1l2*$^{-/-}$ male and female mice at the age of 6 months.

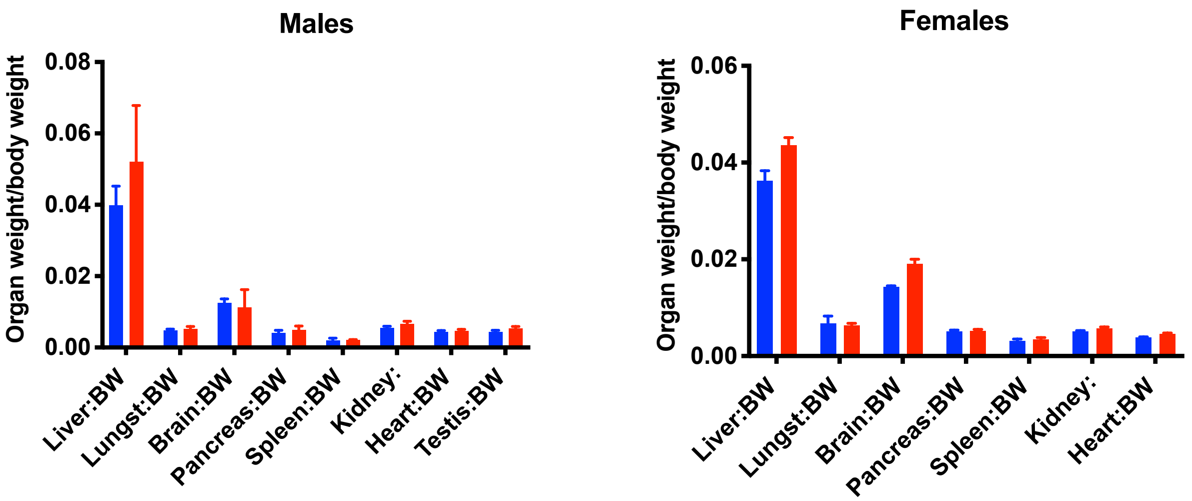

Supplementary Fig. S3. Organ to body weight ratio of *Aldh1l2*$^{+/+}$ and *Aldh1l2*$^{-/-}$ male and female mice at the age of 6 months.

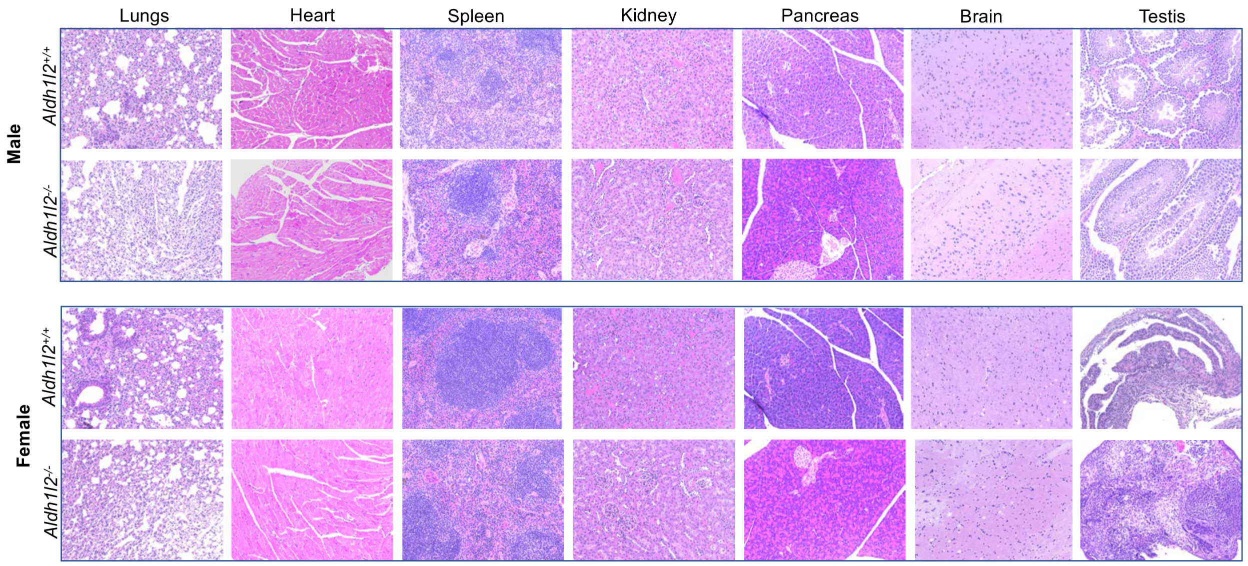

Supplementary Fig. S4. H&E staining of tissue sections from *Aldh1l2*$^{+/+}$ and *Aldh1l2*$^{-/-}$ mice.

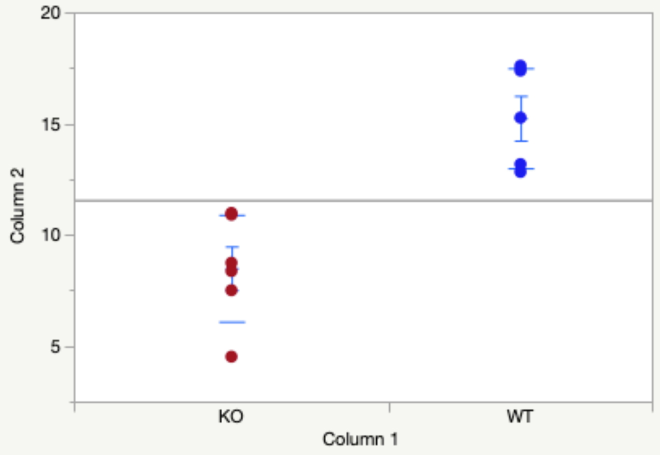

Means and Std Deviations

Level	Number	Mean	Std Dev	Std Err Mean	Lower 95%	Upper 95%
KO	6	8.4987096	2.4006247	0.9800509	5.9794084	11.018011
WT	5	15.245475	2.2492511	1.0058957	12.452661	18.038289

t Test

WT-KO

Assuming unequal variances

Difference	6.74677	t Ratio	4.804036
Std Err Dif	1.40440	DF	8.831834
Upper CL Dif	9.93297	Prob > |t|	0.0010*
Lower CL Dif	3.56056	Prob > t	0.0005*
Confidence	0.95	Prob < t	0.9995

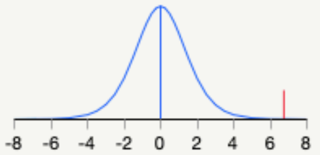

Supplementary Fig. S5. NADPH in liver of *Aldh1l2*$^{+/+}$(WT) and *Aldh1l2*$^{-/-}$(KO) mice measured by a fluorescence kit. 5-6 samples (mice) were analyzed for each genotype. Each sample was measured 4 times (technical replicate). Averages of technical replicates were used to calculate mean ± SE.

NADP Total in Liver

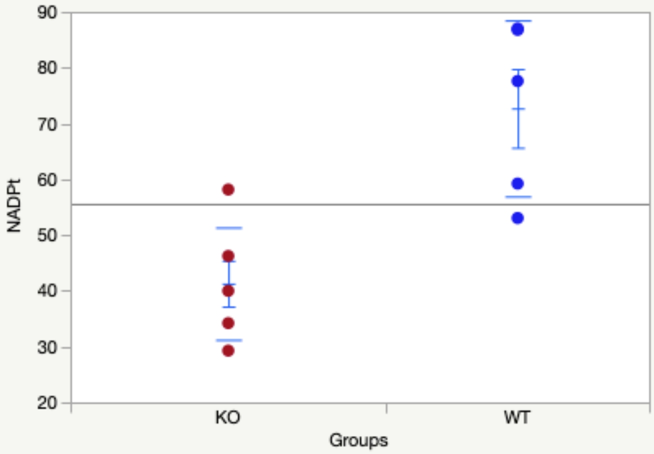

Means and Std Deviations

Level	Number	Mean	Std Dev	Std Err Mean	Lower 95%	Upper 95%
KO	6	41.316365	10.068012	4.1102485	30.750635	51.882095
WT	5	72.736802	15.77734	7.0558408	53.146648	92.326957

t Test

WT-KO

Assuming unequal variances

Difference	31.4204	t Ratio	3.847845
Std Err Dif	8.1657	DF	6.5701
Upper CL Dif	50.9884	Prob > |t|	0.0071*
Lower CL Dif	11.8525	Prob > t	0.0036*
Confidence	0.95	Prob < t	0.9964

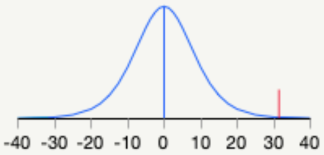

Supplementary Fig. S6. NADP Total (NADPt) in liver of *Aldh1l2*$^{+/+}$(WT) and *Aldh1l2*$^{-/-}$(KO) mice measured by a fluorescence kit. 5-6 samples (mice) were analyzed for each genotype. Each sample was measured 4 times (technical replicate). Averages of technical replicates were used to calculate mean ± SE

NADP/NADPH ratio in liver

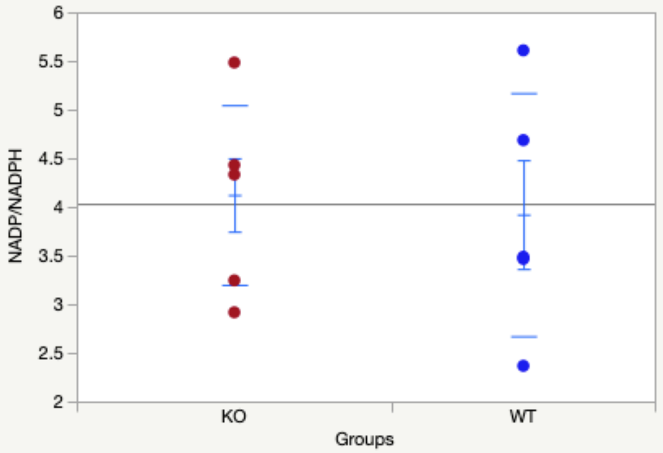

Means and Std Deviations

Level	Number	Mean	Std Dev	Std Err Mean	Lower 95%	Upper 95%
KO	6	4.1240229	0.9233648	0.3769621	3.1550109	5.0930348
WT	5	3.9225057	1.2496863	0.5588767	2.3708152	5.4741962

t Test

WT-KO

Assuming unequal variances

Difference	-0.2015	t Ratio	-0.29893
Std Err Dif	0.6741	DF	7.264615
Upper CL Dif	1.3808	Prob > |t|	0.7734
Lower CL Dif	-1.7839	Prob > t	0.6133
Confidence	0.95	Prob < t	0.3867

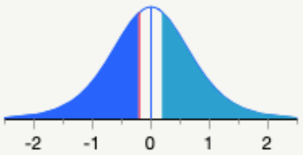

Supplementary Fig. S7. NADP/NADPH in liver of *Aldh1l2*$^{+/+}$(WT) and *Aldh1l2*$^{-/-}$(KO) mice measured by a fluorescence kit. 5-6 samples (mice) were analyzed for each genotype. Each sample was measured 4 times (technical replicate). Averages of technical replicates were used to calculate mean ± SE.

ATP assay in Liver Mitochondria (WT vs KO)

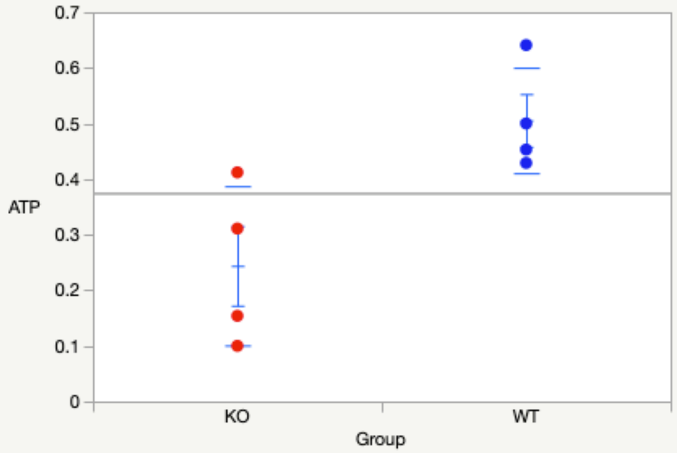

Means and Std Deviations

Level	Number	Mean	Std Dev	Std Err Mean	Lower 95%	Upper 95%
KO	4	0.24425	0.1432349	0.0716175	0.0163312	0.4721688
WT	4	0.50575	0.0948661	0.0474331	0.3547968	0.6567032

t Test

WT-KO

Assuming unequal variances

Difference	0.261500	t Ratio	3.044207
Std Err Dif	0.085901	DF	5.207225
Upper CL Dif	0.479698	Prob > |t|	0.0272*
Lower CL Dif	0.043302	Prob > t	0.0136*
Confidence	0.95	Prob < t	0.9864

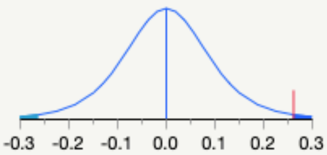

Supplementary Fig S8. Levels of ATP in mitochondria isolated from liver of *Aldh1l2*$^{+/+}$(WT) and *Aldh1l2*$^{-/-}$(KO) mice measured by a colorimetric assay. Four samples (mice) were analyzed for each genotype. Each sample was measured 4 times (technical replicate). Averages of technical replicates were used to calculate mean ± SE.

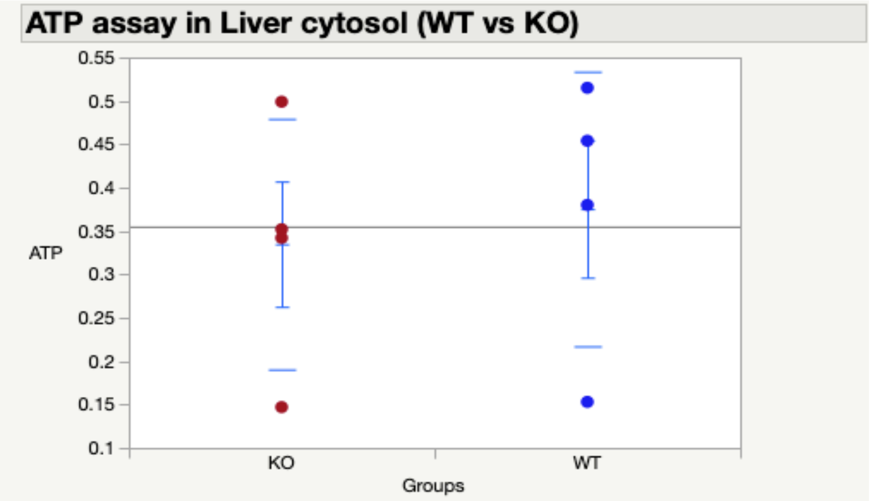

Means and Std Deviations

Level	Number	Mean	Std Dev	Std Err Mean	Lower 95%	Upper 95%
KO	4	0.335	0.1444276	0.0722138	0.1051834	0.5648166
WT	4	0.3755	0.1582709	0.0791354	0.1236557	0.6273443

t Test

WT-KO

Assuming unequal variances

Difference	0.04050	t Ratio	0.378039
Std Err Dif	0.10713	DF	5.950427
Upper CL Dif	0.30317	Prob > |t|	0.7185
Lower CL Dif	-0.22217	Prob > t	0.3593
Confidence	0.95	Prob < t	0.6407

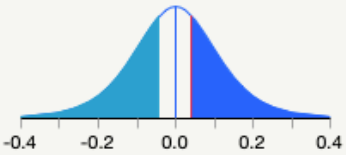

Supplementary Fig S9. Levels of ATP in cytosolic fraction isolated from liver of *Aldh1l2*$^{+/+}$(WT) and *Aldh1l2*$^{-/-}$(KO) mice measured by a colorimetric assay. Four samples (mice) were analyzed for each genotype. Each sample was measured 4 times (technical replicate). Averages of technical replicates were used to calculate mean ± SE.

ATP assay in whole liver lysate (WT vs KO)

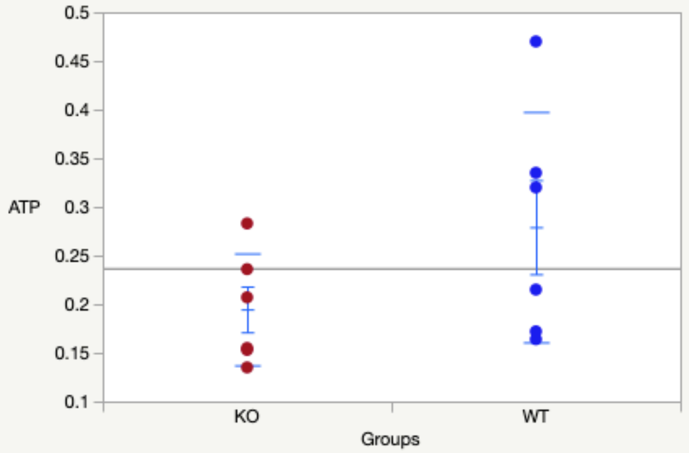

Means and Std Deviations

Level	Number	Mean	Std Dev	Std Err Mean	Lower 95%	Upper 95%
KO	6	0.1948333	0.0575028	0.0234754	0.1344879	0.2551788
WT	6	0.2793333	0.1183785	0.0483278	0.1551027	0.4035639

t Test

WT-KO

Assuming unequal variances

Difference	0.08450	t Ratio	1.572744
Std Err Dif	0.05373	DF	7.235122
Upper CL Dif	0.21071	Prob > |t|	0.1584
Lower CL Dif	-0.04171	Prob > t	0.0792
Confidence	0.95	Prob < t	0.9208

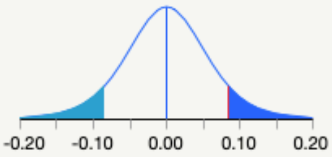

Supplementary Fig S10. Levels of ATP in whole liver lysate isolated from *Aldh1l2*$^{+/+}$(WT) and *Aldh1l2*$^{-/-}$(KO) mice measured by a colorimetric assay. Six samples (mice) were analyzed for each genotype. Each sample was measured 4 times (technical replicate). Averages of technical replicates were used to calculate mean ± SE.

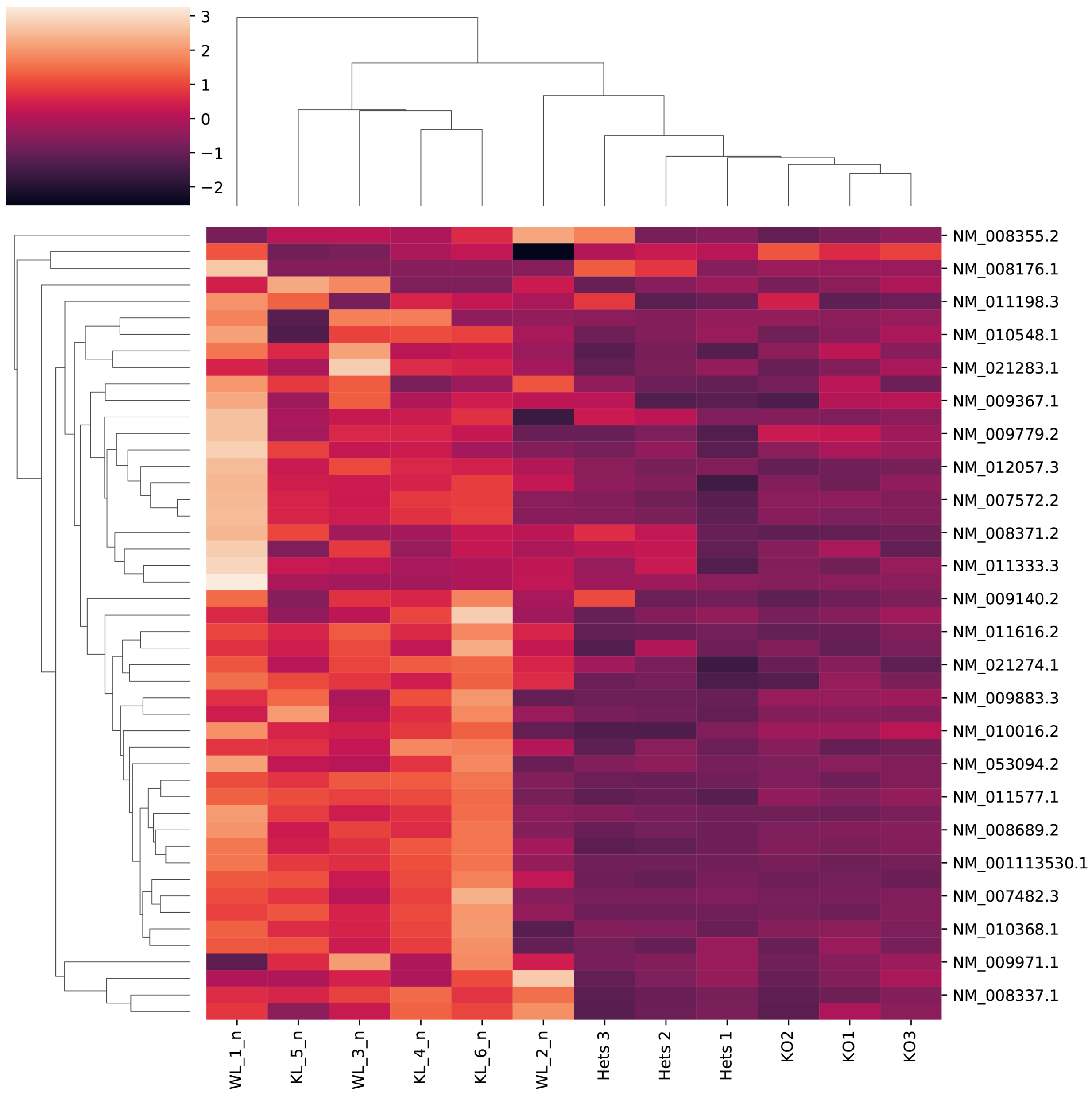

Supplementary Fig S11. Heat map representation of the NanoString data for the four genotypes.

Each protein was standardized so that it has mean 0 and standard deviation 1.

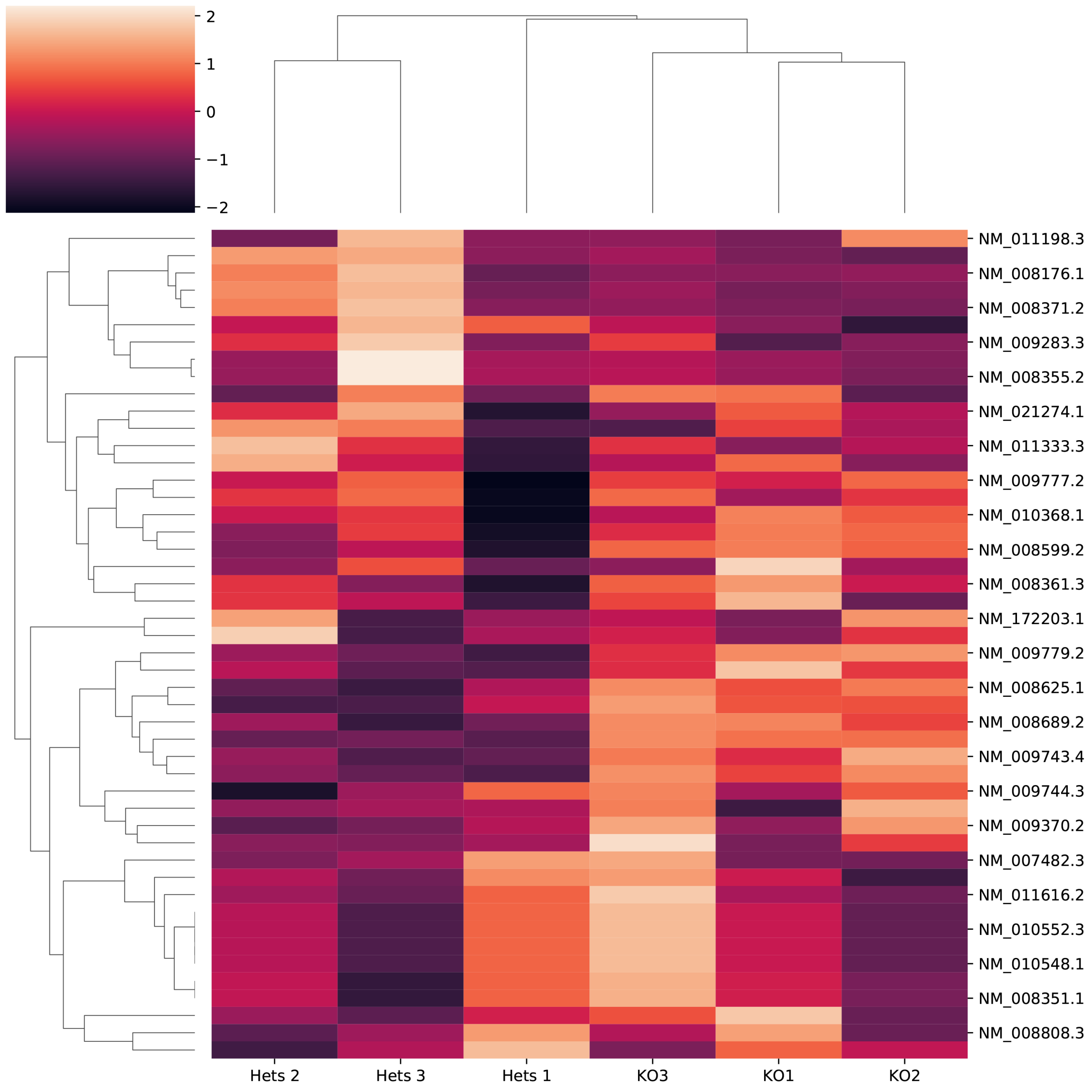

Supplementary Fig S12. Heat map representation of the NanoString data for the *Aldh1l*$^{+/-}$ and *Aldh1l2*$^{-/-}$ genotypes (based on the entire panel of 242 genes).

Supplementary Table S1. Primers used for genotyping.

Primers	Sequence	Amplicon size (bp)
WTf	5′- AATTGGTGGTTCTCTCAAGTCTG -3′	338 bp
WTr	5′- GCACCCATAAAGGGGCTCAAG -3′	
RAF5-L2	5′- CACACCTCCCCCTGAACCTGAAA -3′	598 bp
TTR1-L2	5′- GACATATACTGACCTCTGAGGGTGGC-3′	

Supplementary Table S2. List of primary antibodies used for Western blot assays.

Target	Antibody	Source	Cat# or reference*	Dilution
ALDH1L1	Rabbit polyclonal	In-house	[1]	1:10,000
ALDH1L2	Rabbit polyclonal	In-house	[2]	1:10,000
Hsp60	Rabbit polyclonal	Abcam	ab46798	1:20,000
VDAC	Rabbit monoclonal	Abcam	ab154856	1:1,000
Actin	Mouse monoclonal	Abcam	ab8226	1:10,000

1 Krupenko, S. A. and Oleinik, N. V. (2002) 10-formyltetrahydrofolate dehydrogenase, one of the major folate enzymes, is down-regulated in tumor tissues and possesses suppressor effects on cancer cells. Cell Growth Differ. **13**, 227-236

2 Krupenko, N. I., Dubard, M. E., Strickland, K. C., Moxley, K. M., Oleinik, N. V. and Krupenko, S. A. (2010) ALDH1L2 is the mitochondrial homolog of 10-formyltetrahydrofolate dehydrogenase. J. Biol. Chem. **285**, 23056-23063

Supplementary Table S3. Fecundity, litter size weight and male/female numbers for different combinations of breeder genotypes. Data are expressed as mean ± SE.

Parameter	$M^{+/+}$ X $F^{+/+}$	$M^{-/-}$ X $F^{+/+}$	$M^{+/+}$ X $F^{-/-}$	$M^{-/-}$ X $F^{-/-}$
# of pairs	3	7	6	3
Litter freq/mo	1.4±0.05	1.1±0.05	1.2±0.08	0.95±0..07
Litter size	5.6±0.35	7±0.38	6.1±0.40	6.1±0.59
No of males	34	115	83	61
Weight(g)	14±0.25	13±0.20	12±0.21	13±0.20
No of females	24	74	78	47
Weight(g)	13±0.26	11±0.26	11±0.17	11±.0.18

Supplementary Table S4. Sex and genotype distribution of progeny from intercrosses of *Aldh1l1*$^{+/-}$ mice.

Genotype	Male	Female	Total
Aldh1l2$^{+/+}$	20(29%)	43(35%)	63(32%)
Aldh1l2$^{+/-}$	33(48%)	49(40%)	82(43%)
Aldh1l2$^{-/-}$	16(23%)	31(25%)	47(25%)
Total	69 (100%)	123 (100%)	192 (100%)
